# A proinsulin-dependent interaction between ENPL-1 and ASNA-1 in neurons is required to maintain insulin secretion in *C. elegans*

**DOI:** 10.1242/dev.201035

**Published:** 2023-03-20

**Authors:** Agnieszka Podraza-Farhanieh, Dorota Raj, Gautam Kao, Peter Naredi

**Affiliations:** ^1^ Department of Surgery, Institute of Clinical Sciences, Sahlgrenska Academy, University of Gothenburg, SE413 45 Gothenburg, Sweden; ^2^ Department of Surgery, Sahlgrenska University Hospital, SE413 45 Gothenburg, Sweden

**Keywords:** ER chaperone, Diabetes, Insulin signaling

## Abstract

Neuropeptides, including insulin, are important regulators of physiological functions of the organisms. Trafficking through the Golgi is crucial for the regulation of secretion of insulin-like peptides. ASNA-1 (TRC40) and ENPL-1 (GRP94) are conserved insulin secretion regulators in *Caenorhabditis elegans* (and mammals), and mouse *Grp94* mutants display type 2 diabetes. ENPL-1/GRP94 binds proinsulin and regulates proinsulin levels in *C. elegans* and mammalian cells. Here, we have found that ASNA-1 and ENPL-1 cooperate to regulate insulin secretion in worms via a physical interaction that is independent of the insulin-binding site of ENPL-1. The interaction occurs in DAF-28/insulin-expressing neurons and is sensitive to changes in DAF-28 pro-peptide levels. Consistently, ASNA-1 acted in neurons to promote DAF-28/insulin secretion. The chaperone form of ASNA-1 was likely the interaction partner of ENPL-1. Loss of *asna-1* disrupted Golgi trafficking pathways. ASNA-1 localization to the Golgi was affected in *enpl-1* mutants and ENPL-1 overexpression partially bypassed the ASNA-1 requirement. Taken together, we find a functional interaction between ENPL-1 and ASNA-1 that is necessary to maintain proper insulin secretion in *C. elegans* and provides insights into how their loss might cause diabetes in mammals.

## Teaser

**Summary:** Analysis of worm homologues of the mammalian diabetogenic genes *Grp94* (*enpl-1*) and *Get3* (*asna-1*) shows how they act together to support insulin secretion, suggesting that loss of this interaction might cause diabetes.

## INTRODUCTION

Diabetes mellitus is a chronic disorder characterized by improper glucose homeostasis. Insulin, a hormone synthesized by the pancreatic β-cells, has an essential role in the regulation of growth and metabolism. The underlying cause of the diabetes is related to the insufficient production of insulin or the improper response of the body to the insulin, which lead to increased blood glucose levels (Ashcroft and Rorsman, 2012; DeFronzo et al., 2015). The regulation of insulin secretion is controlled at several levels, including transcription, trafficking, proteolytic processing, and packaging into dense core vesicles and their release (Fu et al., 2013). Although much is known about the pathogenesis and progression of the diabetes, there is still a great need to characterize novel candidates and mechanisms that regulate insulin maturation and secretion. Work in *C. elegans* on the secretion of insulin like peptides has contributed much to our understanding.

ENPL-1 is a *C. elegans* homolog of the endoplasmic reticulum (ER) chaperone GRP94 (also known as GP96 and HSP90B1), which has functions both in ER and non-ER compartments (Frasson et al., 2009; Patel et al., 2013; Podraza-Farhanieh et al., 2020). It positively regulates DAF-28/insulin-like peptide (ILP) secretion at the level of DAF-28 maturation (Podraza-Farhanieh et al., 2020). ENPL-1 is likely required to maintain proper homeostasis in the ER as ER stress markers are upregulated in *enpl-1* mutants (Natarajan et al., 2013). Mammalian GRP94 also helps to maintain proper quality control in the ER of unstressed cells and during the ER-associated degradation (Argon and Simen, 1999; Christianson et al., 2008). The role of GRP94 and its homologs is essential in organismal development as the deletion of the *Drosophila Gp93* gene leads to growth defects (Maynard et al., 2010); *Grp94* knockdown in mice causes embryonic lethality (Mao et al., 2010), and leads to impaired glucose tolerance (Kim et al., 2018), type 2 diabetes (Ghiasi et al., 2019) and defects in the trafficking of the *HER2* (*Erbb2*) oncogene (Patel et al., 2013).

The client set of GRP94 is relatively small and restricted to secreted and membrane proteins such as Toll-like receptors (Liu and Li, 2008; Staron et al., 2010), integrins (Hong et al., 2013), insulin-like growth factors (Barton et al., 2012; Ostrovsky et al., 2009a) and insulin (Ghiasi et al., 2019). Despite the variety of roles mediated by GRP94, very few of its co-chaperones and other proteins that modulate its function have been identified, in contrast to its cytoplasmic paralog HSP90. The first identified co-chaperone that is required for GRP94 function is an ER lumen chaperone, CNPY3 (Liu et al., 2010). GRP94 interacts with CNPY3 to properly fold TLR proteins, which have functions in innate immunity against microbial infections (Kumar et al., 2009; Medzhitov, 2001). Interestingly, the role of CNPY3 has been suggested to support the loading of TLR proteins onto GRP94, indicating for the first time that an ER luminal chaperone requires help from other chaperones to fulfil its function. The other well-described chaperone of GRP94 is BiP (Huang et al., 2022; Jansen et al., 2012; Jin et al., 2021; Sun et al., 2019). GRP94 requires BiP to accelerate its open state and for trapping the proIGF2 client protein. The slow ATPase activity (Frey et al., 2007) and the slow closure of GRP94 (Huang et al., 2022) suggest that GRP94 requires other chaperones to assist in the conformational changes that are required for its binding to client proteins. Clients bind via the defined client-binding domain CBD but the regions of GRP94 required for the co-chaperone association are unknown.

ASNA-1/TRC40/GET3 is a conserved protein whose function has been mainly associated with transporting tail-anchored proteins (TAPs) to the ER (Favaloro et al., 2008; Schuldiner et al., 2008; Stefanovic and Hegde, 2007). Work in yeast and worms shows that ASNA-1/GET3 is found in two redox-sensitive states that have distinct functions and structures (Raj et al., 2021; Voth et al., 2014). Reduced ASNA-1 has a role in inserting TAPs into the ER membrane, whereas the oxidized ASNA-1 is a general chaperone with roles in protecting cells from oxidative damage and aggregated proteins (Powis et al., 2012; Voth et al., 2014). Mutations *TRC40* (*GET3*) in human are associated with diseases such as epilepsy and heart development (Verhagen et al., 2019). Furthermore, loss of *asna-1* causes insulin secretion defects in *C. elegans* (Kao et al., 2007) and loss of *Get3* in pancreatic β cells in mice leads to type 2 diabetes (Norlin et al., 2016, 2018). Although some phenotypes of *asna-1* mutants are associated with the defective TAP insertion, it remains unknown whether the defect in insulin secretion is a consequence of mis-inserted TAPs via its reduced dimeric form or because of loss of functions associated with the oxidized tetrameric form.

Here, we show that ENPL-1 and ASNA-1 work together to mediate the secretion of the DAF-28 ILP. The interaction requires DAF-28/ILP pro-peptide, and increased proinsulin levels promote higher levels of interaction between ASNA-1 and ENPL-1. The ENPL-1/ASNA-1 interaction is more likely to take place when ASNA-1 is oxidized and in its chaperone form. This interaction occurs independently of the DAF-28-binding site in ENPL-1*.* We find that, although both proteins are present in most tissues, most of the interaction between the two proteins occurs in neurons, specifically in neurons that express DAF-28/ILP. We also find that overexpression of ENPL-1 can partially bypass the strict block of DAF-28/ILP secretion in *asna-1* mutants. We demonstrate that, in neurons, ASNA-1 is localized to the Golgi and this localization is defective in *enpl-1* mutants. As ENPL-1 is important for proinsulin binding, we show that the interaction of ENPL-1 and ASNA-1 is necessary to maintain proper insulin secretion in *C. elegans*.

## RESULTS

### ENPL-1 and ASNA-1 interact *in vivo* in intact *C. elegans*

In mouse models, knockdown of the homologs of both *asna-1* and *enpl-1* (*Trc40* and *Grp94*) result in type 2 diabetes (Kim et al., 2018; Norlin et al., 2016). In *C. elegans* both ASNA-1 and ENPL-1 positively promote insulin secretion (Kao et al., 2007; Podraza-Farhanieh et al., 2020), and *enpl-1* was identified in a screen for RNAi clones that produced an *asna-1(-)*-like phenotype (Billing et al., 2012). As a first step to investigate a possible interaction between the two proteins, we asked whether the levels of *asna-1* and *enpl-1* are influenced by the loss of each other. Western blotting and qRT-PCR analysis of *asna-1* levels in *enpl-1(ok1964)* mutants indicated that levels of *asna-1* are unchanged compared with wild type (Fig. S1A,B). On the other hand, qRT-PCR analysis of *enpl-1* in *asna-1(ok938*) mutants showed that levels of *enpl-1* are significantly upregulated (Fig. S1C). Consistently, increased levels of the mouse ENPL-1 homolog GRP94, have been previously reported in *Trc40* knockdown mice (Norlin et al., 2016). Previously we have shown that both *enpl-1* and *asna-1* mutants are sterile (Kao et al., 2007; Podraza-Farhanieh et al., 2020). *enpl-1* mutants from *enpl-1/+* mothers lay dead embryos while adult *asna-1* mutants from *asna-1/+* mothers produce no embryos, and animals depleted for maternal and zygotic *asna-1* arrest at the 1st larval stage (L1). The phenotype of *asna-1(ok938);enpl-1(ok1964)* double mutants was more severe, as no double-mutant larvae were found, indicating that the double mutants displayed embryonic arrest. Specifically, from an *enpl-1(ok1964)/tmC5(tmIs1220); asna-1(ok938)/oxTi719 unc-32(e189)* strain, a total of 1593 double heterozygotes were counted, indicating that the total population examined was 3186 worms. One-sixteenth of those (i.e. 198 animals) are expected to be *asna-1;enpl-1* double mutants. However, no larvae or adults of this class, which lack both balancers, were observed. The ‘missing class’ indicates that all *asna-1;enpl-1* double mutants likely died as embryos.

We next asked what the consequence would be of depleting ASNA-1 in *enpl-*1 mutants after the L1 stage. To do this, we used the auxin-mediated degradation system using a strain containing an allele of *asna-1(syb2249)* in which the AID (auxin-dependent degron) tag and mNeonGreen were inserted in the gene, and the *Arabidopsis* TIR1 ubiquitin ligase protein was expressed in all somatic cells using the *ieSi57* transgene. This system will produce rapid degradation of the AID tagged protein when placing the worms on auxin-containing agar plates at any developmental stage. We found that 4th larval stage *enpl-1(ok1964);asna-1(syb2249);ieSi57* worms exposed to auxin did not produce any embryos, showing that the maternal effect sterile phenotype of *enpl-1* mutants was modified by the depletion of ASNA-1 (Fig. S1D).

**Fig. 1.**
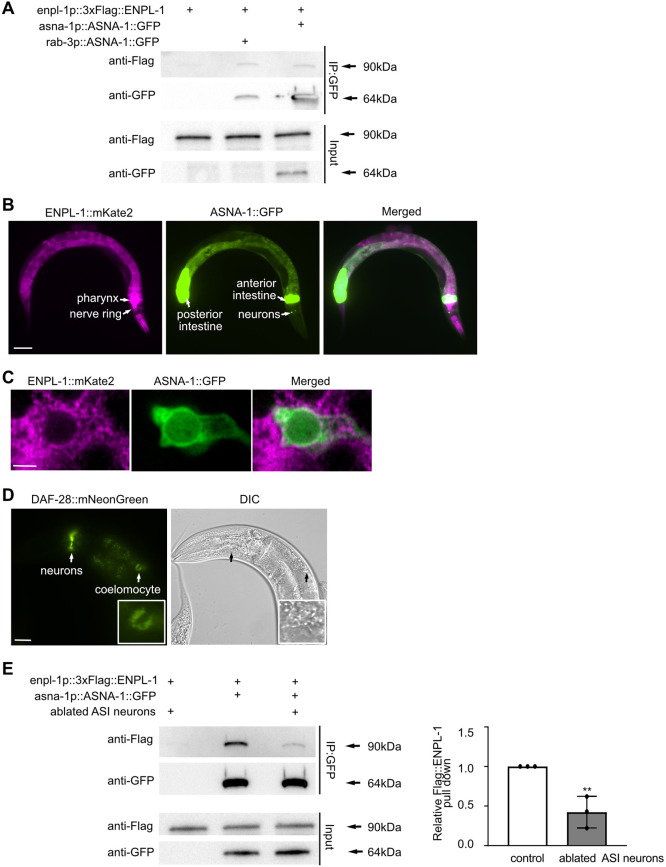
**ENPL-1 and ASNA-1 interact in *C. elegans* in insulin-expressing neurons.** (A) Co-immunoprecipitation experiments with anti-GFP affinity beads from lysates of adult animals expressing 3xFlag::ENPL-1, co-expressing 3xFlag::ENPL-1+rab3p::ASNA-1::GFP, and 3xFlag::ENPL-1+asna-1p::ASNA-1::GFP. Inputs from every strain used in the analysis were used as a loading control. (B) Representative fluorescence microscopy images of an adult worm expressing ENPL-1::mKate2 and ASNA-1::GFP (*n*≥12). White arrows indicate structures where ENPL-1::mKate2 and ASNA-1::GFP are present. Scale bar: 40 µm. (C) Representative confocal images of an ASI neuron from an adult worm expressing ENPL-1::mKate2 and ASNA-1::GFP (*n*≥12). Scale bar: 4 µm. (D) Representative fluorescence microscopy and differential interference contrast (DIC) microscopy images of adult worms expressing DAF-28::mNeonGreen. Arrows indicate locations of expression and secretion of DAF-28::mNeonGreen. Scale bar: 20 µm. Insets show magnification of an anterior coelomocyte. (E) Co-immunoprecipitation experiments with anti-GFP affinity beads from lysates of adult animals expressing 3xFlag::ENPL-1 or co-expressing 3xFlag::ENPL-1+ASNA-1::GFP with and without *oyIs84* (ablated ASI neurons) followed by western blot analysis. Inputs from every strain used in the analysis were used as a loading control. The experiment was performed in triplicate. Quantification shows relative levels of 3xFlag::ENPL-1 immunoprecipitation in the indicated strains. Statistical significance was determined using a two-tailed *t*-test (**P<0.01). Data are mean±s.d.

We then wanted to determine whether the two proteins might interact to promote insulin secretion function and carried out co-immunoprecipitation followed by western blot analysis in strains expressing a multi-copy transgene of ASNA-1::GFP and a single copy of 3xFlag::ENPL-1. Both tagged proteins were expressed under their native promoters (Kao et al., 2007; Podraza-Farhanieh et al., 2020). This analysis revealed that the two proteins can physically bind (Fig. 1A, Fig. S2A). To account for the possibility that the binding between these two proteins occurred after preparation of the lysates, we expressed ASNA-1::GFP under the control of a pan-neuronal promoter and 3xFlag::ENPL-1 under the control of a body wall muscle promoter, and carried out the co-immunoprecipitation followed by western blot analysis. The results obtained from this experiment indicated that there is no post-lysis interaction as the two proteins do not co-immunoprecipitate when expressed in separate tissues (Fig. S3A). The antibodies used were highly specific: the anti-Flag antibody detected 3xFlag::ENPL-1 but not ASNA-1::GFP; the anti-GFP antibody detected ASNA-1::GFP but not 3xFlag::ENPL-1 (Fig. S3B).

### ENPL-1 and ASNA-1 interact in the DAF-28/ILP-expressing ASI neuron

As both ENPL-1 and ASNA-1 are required for DAF-28 secretion, we wanted to know whether the binding between these two proteins occurred in DAF-28-expressing cells. The DAF-28 protein is expressed in only two neurons: ASI and ASJ (Kao et al., 2007). We expressed ASNA-1::GFP under the *prab3* pan-neuronal promoter and performed co-immunoprecipitation experiments in worms co-expressing 3xFlag::ENPL-1 driven by its own promoter. This analysis showed that the same amount of 3xFlag::ENPL-1 was co-immunoprecipitated when ASNA-1::GFP was expressed under the neuron-specific promoter when compared with expression using its own promoter. This indicated that the two proteins interacted in neurons of *C. elegans* and that the bulk of binding occurred in neurons (Fig. 1A, Fig. S2A). ASNA-1::GFP from the *svIs56* transgene is expressed in two pairs of head neurons, ASI and ASK, and in the intestine (Kao et al., 2007), whereas ENPL-1::mKate2 is widely expressed in the whole animal, including the pharynx and neurons (Podraza-Farhanieh et al., 2020) (Fig. 1B). ASNA-1 and ENPL-1 expression was found in the overlapping neuron ASI based on confocal microscopy of worms co-expressing ASNA-1::GFP and ENPL-1::mKate2 (Fig. 1C). We have previously showed that DAF-28::GFP expressed from the multi-copy transgene *svIs69*, is detected in ASI and ASJ neurons, and in the intestine. The secreted DAF28:GFP is taken up by the coelomocytes sitting in the psedocoelomic space (Kao et al., 2007). To determine whether this transgene was accurately reporting the expression of DAF-28, we analyzed a strain *daf-28(syb3050)*, in which the mNeonGreen gene was inserted immediately before the stop codon in the genomic locus in order to analyze DAF-28 expression and secretion without overexpression. Our analysis showed that the expression of DAF-28::mNeonGreen was still found in only two pairs of head neurons (both in axons and cell bodies), and the protein was secreted into the pseudocoelom and taken up by the coelomocytes from the 4th larval stage onwards (Fig. 1D, Fig. S4A,B). No expression was detected in the intestine or in other tissues. To investigate whether the interaction between ASNA-1 and ENPL-1 occurred in DAF-28-expressing neurons, we examined the consequence of killing the ASI neurons (Beverly et al., 2011) on the ability of ENPL-1 and ASNA-1 to bind to each other. We found that the immunoprecipitation of 3xFlag::ENPL-1 by ASNA-1::GFP was significantly reduced when ASI neurons were killed (Fig. 1E, Fig. S2B). Confocal microscopy analysis showed that the two proteins are co-expressed in the same neurons (Fig. 1C). Taken together, this analysis indicated that ASNA-1 and ENPL-1 interacted in *C. elegans*, and that the interaction took place in ASI neurons, which are also a site of DAF-28 expression. We concluded that, although both ENPL-1 and ASNA-1 were widely expressed, their interaction might be happening to a large extent in only DAF-28-expressing cells.

### ASNA-1 function is required in neurons to regulate insulin secretion

**Fig. 2.**
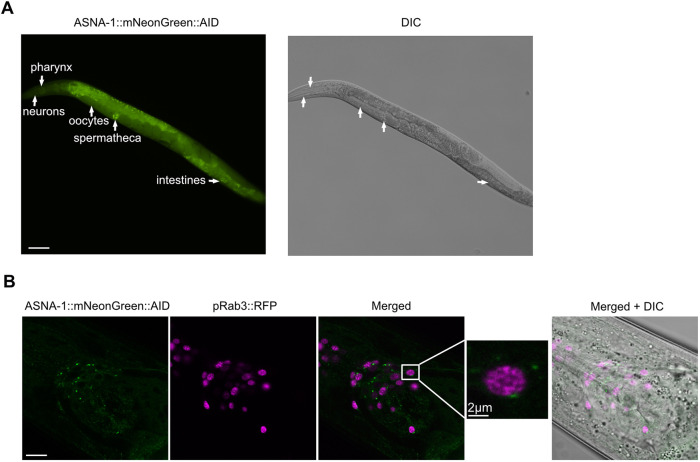
**ASNA-1::mNeonGreen::AID is found in the Golgi of neurons.** (A) Representative fluorescence and differential interference contrast (DIC) images of worms expressing ASNA-1::mNG::AID. White arrows indicate different places where ASNA-1::mNeonGreen::AID is detected, such as the pharynx, neurons, oocytes, spermatheca and the intestine. Scale bar: 20 µm. (B) Representative confocal and differential interference contrast (DIC) images of adult worms expressing Prab-3::NLS::TagRFP (*otIs356*) and ASNA-1::mNeonGreen::AID (*n*≥10). Scale bar: 10 µm. A 5× magnified merged image is provided to indicate the markers in more detail.

**Fig. 3.**
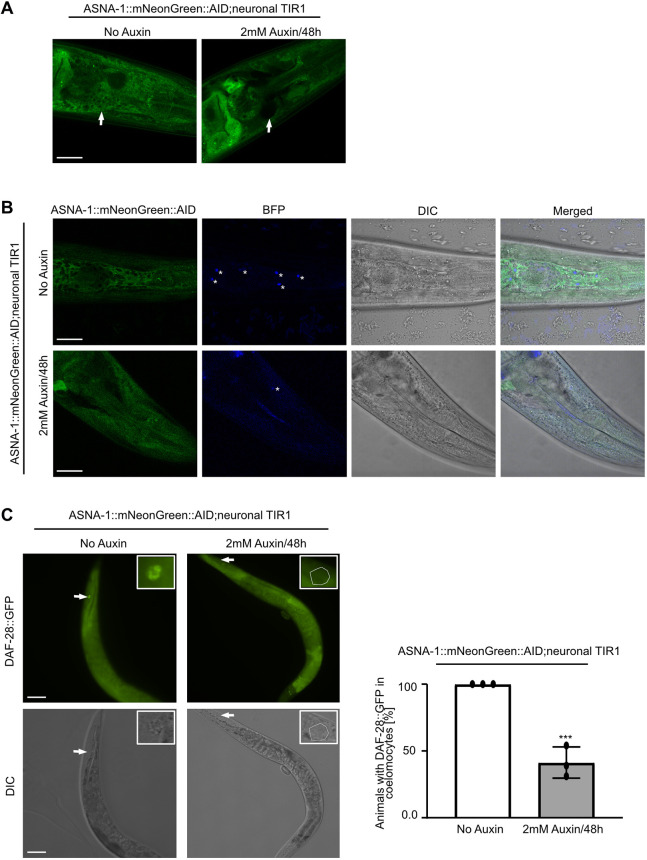
**ASNA-1 is required in neurons to regulate insulin secretion.** (A) Representative confocal images of adult worms expressing ASNA-1::mNG::AID*(syb2249)*;neuronal TiR1::F2A::BFP::AID (from *reSi7*) without auxin or exposed to 2 mM auxin for 48 h. Arrows indicate the position of the nerve ring where ASNA-1 is localized (*n*≥14). Scale bar: 10 µm. (B) Representative confocal and differential interference contrast (DIC) images of adult worms expressing ASNA-1::mNG::AID;neuronal TiR1::F2A::BFP::AID without auxin or exposed to 2 mM auxin for 48 h. Blue fluorescence protein (BFP::AID) was used to show the efficiency of auxin-mediated degradation. Asterisks indicate BFP-positive neurons (*n*≥14). BFP::AID is degraded when worms are exposed to auxin. Scale bars: 10 µm. (C) Representative fluorescence and differential interference contrast (DIC) images of worms expressing ASNA-1::mNG::AID;neuronal TiR1 with DAF-28::GFP without auxin exposure or exposed to 2 mM auxin for 48 h. Arrows indicate the localization of a posterior coelomocyte in each animal. The dashed line outlines a DAF-28::GFP-negative coelomocyte (*n*≥15). Scale bars: 40 µm. The experiments were performed in triplicate. Quantification shows the percentage of animals with DAF-28::GFP in posterior coelomocytes. Statistical significance was determined using a two-tailed *t*-test (***P<0.001). Data are mean±s.d.

Having determined that ENPL-1 and ASNA-1 interact in DAF-28-expressing neurons, we next wanted to investigate whether ASNA-1 function is required in neurons to promote insulin secretion. To this end, we used a strain in which we depleted the ASNA-1 protein specifically from neurons, using the auxin-mediated protein-degradation system (Ashley et al., 2021; Zhang et al., 2015). We used the *asna-1* allele *syb2249* (already described) in which mNeonGreen and AID (auxin-induced degron) were inserted in the last codon of the of the gene. ASNA-1::mNeonGreen::AID (*syb2249*) was widely expressed in the soma and germline, including neurons, pharynx, intestines, oocytes and spermatheca (Fig. 2A). To confirm the neuronal expression, we analyzed worms co-expressing a pan-neuronal nuclear localized tagRFP and ASNA-1::mNeonGreen::AID, and found that ASNA-1::mNeonGreen::AID was expressed in many neurons (Fig. 2B). To deplete ASNA-1::mNeonGreen::AID from neurons, we crossed in the *reSi7* transgene that restricts auxin-mediated depletion of AID-tagged proteins to only neurons (Ashley et al., 2021; Zhang et al., 2015). Depletion using 2 mM auxin for 48 h from L4 stage onwards resulted in significant depletion of blue fluorescent protein (BFP:AID) expressed from *reSi7*. BFP:AID depletion serves as an indicator of effective auxin-mediated degradation. (Fig. 3B). The same conditions also resulted in depletion of ASNA-1::mNeonGreen::AID from neurons (Fig. 3A). Next, to understand whether the neuronal ASNA-1 was responsible for insulin secretion, we analyzed *ASNA-1::mNeonGreen::AID;reSi7;DAF-28::GFP* worms after auxin treatment. Depletion of ASNA-1 from neurons significantly decreased DAF-28::GFP/ILP secretion (Fig. 3C). Taken together, these data indicate that neuronal ASNA-1 is required for DAF-28/ILP secretion in *C. elegans*.

### The interaction between ENPL-1 and ASNA-1 requires the DAF-28 pro-peptide

**Fig. 4.**
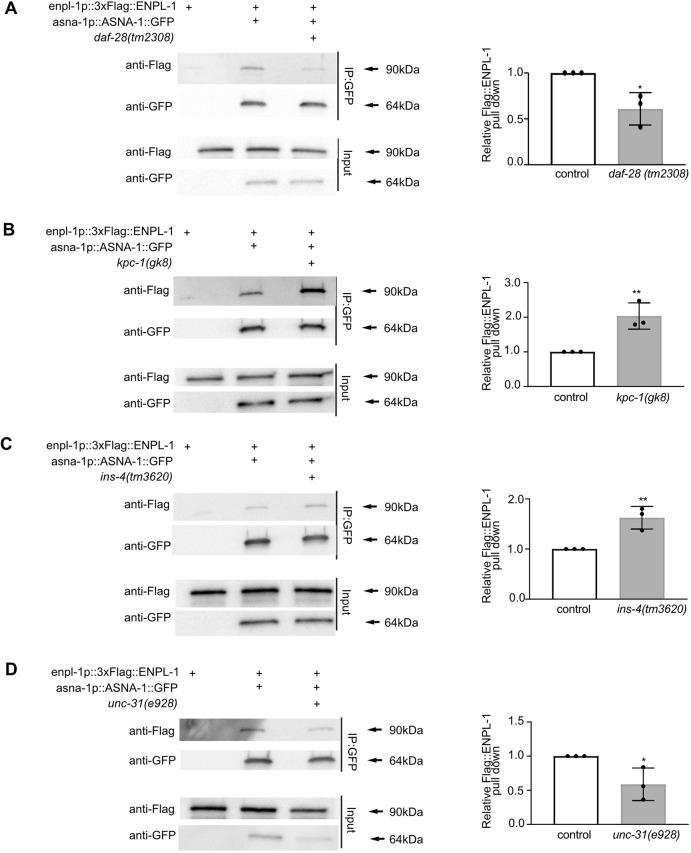
**The ENPL-1/ASNA-1 interaction requires the DAF-28 pro-peptide.** Co-immunoprecipitation experiments with anti-GFP affinity beads from lysates of adult animals expressing 3xFlag::ENPL-1 or co-expressing 3xFlag::ENPL-1+ASNA-1::GFP (A) in *daf-28(tm2308)* mutants, (B) in *kpc-1(gk8)* mutants, (C) in *ins-4(tm3620)* mutants and (D) in *unc-31(e928)* mutants, followed by western blot analysis. Inputs from every strain used in the analysis were used as a loading control. The experiments were performed in triplicate. Quantification shows the relative levels of 3xFlag::ENPL-1 immunoprecipitation in the indicated strains. Statistical significance was determined using a two-tailed *t*-test (*P<0.05, **P<0.01). Data are mean±s.d.

To understand further whether the interaction between ASNA-1 and ENPL-1 was required for DAF-28/ILP secretion, we tested whether DAF-28 was essential for complex formation. We have shown previously that ENPL-1 interacts with DAF-28 pro-peptide via its client-binding domain. The interaction was essential for the processing of proinsulin to mature insulin because only the DAF-28 pro-peptide was detected in *enpl-1* mutants (Podraza-Farhanieh et al., 2020). Consistent with our findings, it has been shown that murine GRP94 is essential for proinsulin handling and is required for insulin secretion (Ghiasi et al., 2019). Mindful of these findings, we asked whether the interaction between ASNA-1 and ENPL-1 could be affected by the lack of DAF-28/ILP. To do this, we carried out co-immunoprecipitation analysis in *daf-28(tm2308)* loss-of-function mutants (Hung et al., 2014) and found that the interaction between ASNA-1 and ENPL-1 was significantly reduced in DAF-28 mutants (Fig. 4A, Fig. S5A). This indicated that the known ENPL-1 client DAF-28 was also required for the proper formation of the ASNA-1/ENPL-1 complex. In order to be properly processed and secreted, proinsulin needs to be cleaved in the dense core vesicles by the proprotein convertases 1/3 and 2 (Rhodes and Alarcón, 1994). *C. elegans* insulin-like peptides also require the activity of proprotein convertases: AEX-5, BLI-4, KPC-1 and EGL-3 (Kass et al., 2001; Thacker and Rose, 2000; Thacker et al., 2000). The DAF-28 pro-peptide is cleaved only by KPC-1 prohormone convertase, whereas INS-4 is cleaved by the EGL-3 prohormone convertase (Hung et al., 2014; Podraza-Farhanieh et al., 2020). As the loss of DAF-28 decreased the interaction between ASNA-1 and ENPL-1, we asked whether there would be any change in the interaction in *kpc-1* mutants, which accumulate DAF-28 pro-peptide (Hung et al., 2014; Podraza-Farhanieh et al., 2020). Co-immunoprecipitation analysis showed that the interaction between ASNA-1::GFP and 3xFlag::ENPL-1 was significantly higher in the *kpc-1* mutants (Fig. 4B, Fig. S5B). We asked next whether another insulin, INS-4, which is processed by EGL-3 prohormone convertase, similarly affected complex formation by performing the co-immunoprecipitation analysis in *ins-4(tm3620)* mutants. In contrast to the findings with *daf-28* mutants, there was no decrease in the immunoprecipitation of ENPL-1 by ASNA-1 pulldown (Fig. 4C, Fig. S6A). We asked further whether the decreased insulin secretion in *C. elegans* might affect the strength of binding between ASNA-1 and ENPL-1. For this, we used *unc-31(e928)* mutants, which display decreased insulin secretion because the UNC-31/CAPS is required for dense-core vesicle release (Speese et al., 2007). In *unc-31* mutants, there is an accumulation of mature DCVs containing fully processed insulin protein (Hammarlund et al., 2008). Co-immunoprecipitation analysis revealed that, in *unc-31* mutants, the interaction between ASNA-1 and ENPL-1 was significantly decreased (Fig. 4D. Fig. S6B). Taken together our analysis showed that DAF-28, and specifically the DAF-28 pro-peptide, is required for efficient complex formation between ASNA-1 and ENPL-1, and levels of the complex were sensitive to the levels of the DAF-28 pro-peptide.

### Increasing levels of oxidized ASNA-1 leads to more interaction with ENPL-1

It has been shown that *C. elegans* ASNA-1 and its yeast homolog GET3 can work as ATPase-dependent targeting proteins. However, under the high oxidative stress condition, the proteins undergo an oxidation-dependent conformational change and are converted into an ATPase-independent chaperone (Raj et al., 2021, 2022; Voth et al., 2014).

**Fig. 5.**
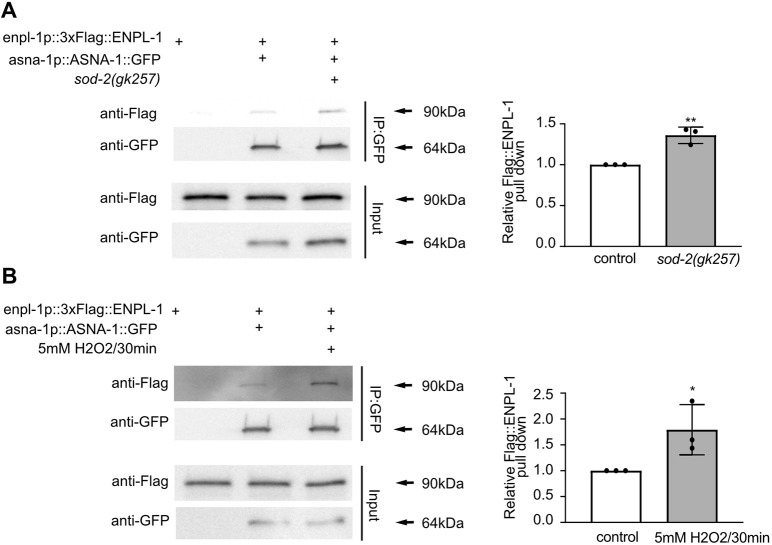
**Conditions promoting ASNA-1 oxidation increase ASNA-1/ENPL-1 complex formation.** (A,B) Co-immunoprecipitation experiments with anti-GFP affinity beads from lysates of adult animals expressing 3xFlag::ENPL-1 or co-expressing 3xFlag::ENPL-1+ASNA-1::GFP (A) in *sod-2(gk257)* mutant or (B) exposed to 5 mM H_2_O_2_ for 30 min, followed by western blot analysis. Inputs from every strain used in the analysis were used as a loading control. The experiments were performed in triplicate. Quantification shows the relative levels of 3xFlag::ENPL-1 immunoprecipitation in the indicated strains. Statistical significance was determined using a two-tailed *t*-test (*P<0.05, **P<0.01). Data are mean±s.d.

To understand further the characteristics of the interaction between ASNA-1 and ENPL-1, we asked whether conditions that produce high ROS levels and convert ASNA-1 to the oxidized form could influence the interaction with ENPL-1. *sod-2(gk257)* mutants or exposure to H_2_O_2_, are sufficient to increase levels of oxidized ASNA-1::GFP at the expense of the reduced form of the protein (Raj et al., 2021). We performed co-immunoprecipitation in both settings, *sod-2(gk257)* mutants or after exposure to H_2_O_2_, and found a significant increase in the interaction between ASNA-1 and ENPL-1 in both cases (Fig. 5A,B, Fig. S7A,B). We concluded that conditions that promote the conversion of ASNA-1 into the oxidized chaperone form also promoted increased complex formation, indicating that most likely it is the oxidized form of ASNA-1 that interacts with ENPL-1.

### ASNA-1 interacts with ENPL-1 independently of its client-binding domain and does not interact with proinsulin

Given our finding that the chaperone form of ASNA-1 was the likely binding partner of ENPL-1, we next asked whether ASNA-1 had the characteristics of an ENPL-1 client protein or whether its function was instead based on a non-client role. To address this, we used a previously described 3xFlag::ENPL-1 variant, 3xFlag::ENPL-1^ΔCBD^, which has a deletion in the highly conserved client-binding domain (CBD). This deletion was shown to be sufficient to prevent its interaction with the DAF-28/ILP pro-peptide, indicating that the proinsulin is likely a client protein of ENPL-1 (Podraza-Farhanieh et al., 2020). On performing co-immunoprecipitation in worms co-expressing ASNA-1::GFP and 3xFlag::ENPL^ΔCBD^, we found that the interaction of 3xFlag::ENPL-1 with ASNA-1::GFP did not require the CBD, indicating that ASNA-1 was likely not a client protein to ENPL-1, but rather that this interaction requires other domains of ENPL-1 (Fig. S8A). This finding was consistent with the fact that the client set of GRP94 is restricted to secreted and transmembrane proteins. We next asked whether ASNA-1::GFP could immunoprecipitate the DAF-28 pro-peptide. To determine this, we performed a co-immunoprecipitation experiment from worms expressing ASNA-1::GFP and Ollas::DAF-28::MYC (doubled tagged DAF-28/ILP) (Podraza-Farhanieh et al., 2020) but did not detect any interaction between ASNA-1::GFP and DAF-28 pro-peptide (Fig. S8B). We conclude that it is unlikely that there is a direct interaction between ASNA-1 and DAF-28.

### Overexpression of ENPL-1 bypasses the need for ASNA-1 in DAF-28 secretion

**Fig. 6.**
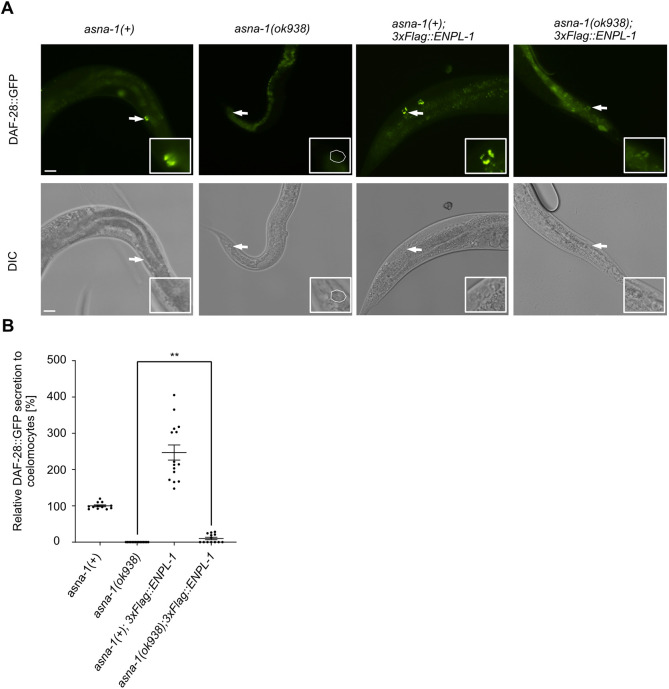
**Overexpression of ENPL-1 bypasses the requirement of ASNA-1 for DAF-28::GFP secretion.** (A) Representative fluorescence and differential interference contrast (DIC) images of adult *asna-1(+)* and *asna-1(ok938)* animals with and without 3xFlag::ENPL-1. White arrows indicate the posterior coelomocytes accumulating GFP in each animal. The dashed line outlines a DAF-28::GFP-negative coelomocyte (*n*≥12). Scale bars: 10 µm. Insets show magnifications of individual coelomocytes. (B) Quantification shows relative DAF-28::GFP fluorescence in posterior coelomocytes, normalized to the *asna-1(+)* control. Data are mean±s.e.m. (*n*≥12). Statistical significance between *asna-1(ok398)* and *asna-1(ok938);3xFlag::ENPL-1* was determined using a Mann–Whitney test (**P<0.01).

*asna-1* has an essential role in promoting insulin secretion and insulin signaling in *C. elegans* as DAF-28::GFP was not secreted in *asna-1(ok938)* mutants (Kao et al., 2007). Knowing that ASNA-1 and ENPL-1 interact *in vivo*, and that the interaction is insulin dependent, we asked whether overexpression of ENPL-1 could modify the insulin secretion defect of *asna-1(ok938)* mutants. Overexpression of ENPL-1 from 3xFlag::ENPL-1 transgene increases insulin secretion (Podraza-Farhanieh et al., 2020). We crossed 3xFlag::ENPL-1 into *asna-1(ok938)* mutants and found that overexpression of ENPL-1 partially bypassed the *asna-1*-mediated block in insulin secretion because we observed increased DAF-28::GFP secretion and uptake by coelomocytes (Fig. 6A,B). This analysis indicated that there was a compensation of function between these two proteins and that increased levels of ENPL-1 partially suppressed the *asna-1(ok938)* mutant defect.

### Loss of *asna-1* perturbs pathways related to ER and Golgi trafficking and transport

**Fig. 7.**
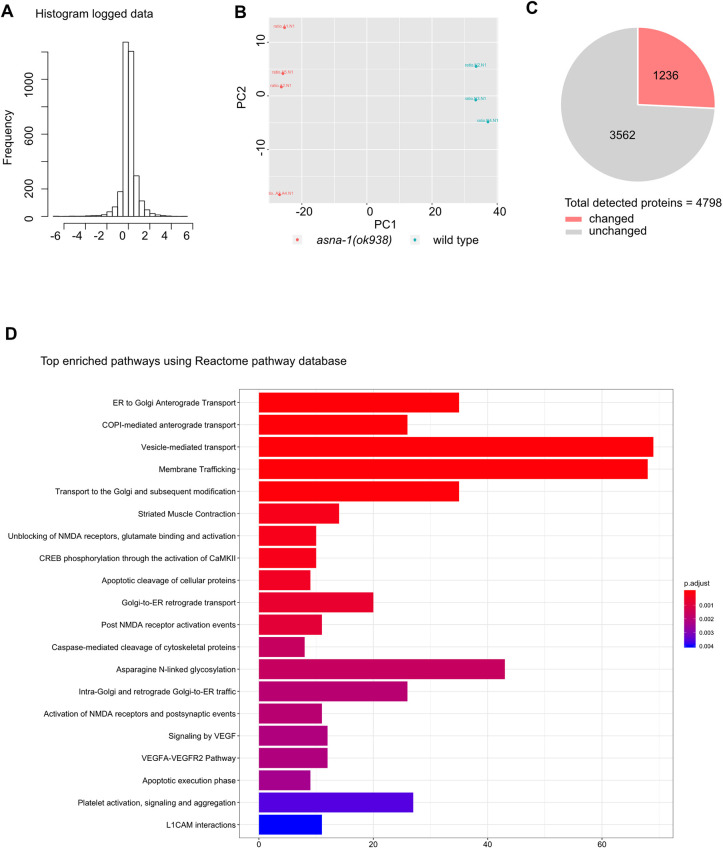
**Quantitative proteomic analysis of *asna-1* mutants showing changes in pathways related to ER and Golgi trafficking and transport.** (A) Histogram data of samples used for global proteomic analysis. (B) Principal component analysis (PCA) of proteomics datasets from 1-day-old adult wild-type animals and *asna-1(ok938)* mutants. (C) Pie chart indicating the number of hits obtained. Gray represents the number of unchanged proteins in the *asna-1(ok938)* mutants; red represents the number of significantly changed proteins in the *asna-1(ok938)* mutants. (D) The Reactome pathway enrichment analysis of differentially expressed proteins in *asna-1(ok938)* mutants. The list shows the top 20 pathways and includes pathways that are either upregulated or downregulated in the *asna-1(ok938)* mutants (FDR<0.01).

To further understand why the lack of ASNA-1 causes insulin secretion defects in animals, we performed a quantitative proteomic analysis of *asna-1(ok938)* mutants and wild-type animals to detect proteins and pathways that were the most affected by the loss of *asna-1*. Principal component analysis (PCA) indicated distinct expression profiles of proteins in wild-type animals compared with *asna-1(ok938)* mutants (Fig. 7A,B). We detected a total of 4798 proteins among which 1236 were significantly changed (FDR<0.01) (Fig. 7C, Table S1). To further understand which pathways were the most affected in the absence of *asna-1*, we performed a Reactome enrichment analysis and examined the top 20 affected pathways (Fig. 7D). Among them were many pathways related to ER and Golgi transport and trafficking, COP I transport and retrograde transport. Taken together, these data shows that the main role of ASNA-1 is at the level of ER and Golgi trafficking.

### ASNA-1 is localized to the Golgi and loss of ENPL-1 affects this distribution

**Fig. 8.**
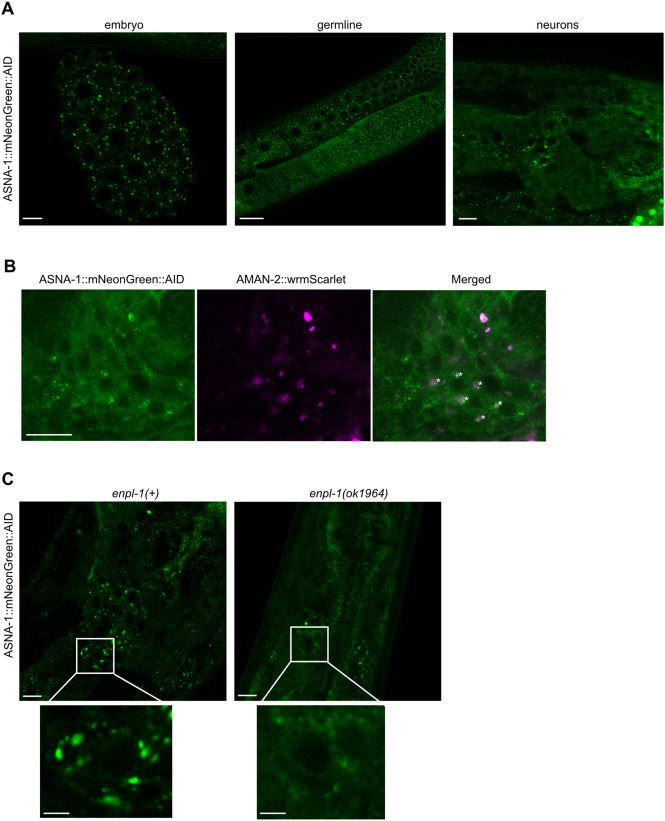
**Loss of *enpl-1* causes defects in localization of ASNA-1::mNeonGreen::AID.** (A) Representative confocal images of adult worms expressing ASNA-1::mNeonGreen::AID in puncta in embryos, germlines and neurons. Scale bars: 5 µm (left and right); 20 µm (middle). (B) Representative confocal images of adult worms expressing ASNA-1::mNeonGreen::AID and AMAN-2::wrmScarlet, *n*≥12. Scale bar: 10 µm. (C) Representative confocal images of adult worms expressing ASNA-1::mNeonGreen::AID in *enpl-1(+)* and in *enpl-1(ok1964)* mutants. Scale bars: 5 µm (2 µm in higher magnifications). *n*≥10 examined for each genotype.

The proteomic analysis indicated that ASNA-1 might affect the Golgi function (Fig. 7D). It has been shown as well that the Golgi morphology was affected in the ASNA1 knockdown mice and led to the formation of small distended membrane stacks (Norlin et al., 2018). The Golgi markers AMAN-2 and SQV-8 localize to Golgi puncta in ASI neurons (Broekhuis et al., 2013). Confocal analysis showed that ASNA-1 was widely expressed in embryos, the germline and in neurons. In all these tissues, ASNA-1::mNeonGreen::AID is observed in puncta that resemble Golgi bodies (Fig. 8A). The punctate distribution of ASNA-1 in neurons was found to be in the Golgi, as ASNA-1:mNeonGreen colocalized with AMAN-2::wrmScarlet expressed in head neurons from the *rawEx90* transgene (Fig. 8B). This punctate localization was disrupted by the loss of *enpl-1*, because in the *enpl-1(ok1964)* mutants we observed ASNA-1::mNeonGreen::AID in a more diffused pattern (Fig. 8C). We conclude that ASNA-1 is found in the Golgi and its localization there depends on ENPL-1.

## DISCUSSION

Here, we provide the evidence that ENPL-1, the homolog of well-known ER chaperone GRP94/HSP90B1, interacts *in vivo* with ASNA-1. Both ASNA-1 and ENPL-1 are expressed in the neurons where the DAF-28/ILP is also expressed (Kao et al., 2007; Podraza-Farhanieh et al., 2020). Our immunoprecipitation analysis showed that ASNA-1 and ENPL-1 interact in neurons. Most of the interaction occurred in ASI neurons and the interaction required the presence of DAF-28. In *kpc-1* mutants with high levels of DAF-28 pro-peptide (Podraza-Farhanieh et al., 2020), we found an increased interaction between ASNA-1 and ENPL-1, indicating that the proinsulin form of DAF-28 likely drives increased binding. Conditions that increased oxidation of ASNA-1 increased the binding between ASNA-1 and ENPL-1, indicating that the oxidized form of ASNA-1, which acts as a chaperone (Voth et al., 2014), is the form that binds to ENPL-1. Our study showed that the client-binding domain of ENPL-1, via which it binds to DAF-28 pro-peptide, was not needed for ASNA-1 binding, as the pull-down ability was not affected when the CBD was mutated. Furthermore, although ENPL-1 formed a complex with DAF-28 pro-peptide, an ASNA-1/DAF-28 pro-peptide complex was not detected, indicating that these two interactions with ENPL-1 are likely occurring separately.

Numerous studies have shown that GRP94 is an important ER chaperone that binds to and ensures the folding of essential client proteins, such as toll-like receptors, proinsulin or insulin-like growth factor 2 (Ghiasi et al., 2019; Liu et al., 2010; Ostrovsky et al., 2009a; Podraza-Farhanieh et al., 2020). However, it remains unknown how many non-client proteins are needed by GRP94 to maintain its proper function. The ATPase activity of GRP94 is relatively low, indicating that GRP94 requires a co-chaperone to enhance its binding to the designated clients (Frey et al., 2007; Ostrovsky et al., 2009b). Recent studies have shown that BiP is a novel interacting partner of GRP94 and that BiP assists with the acceleration of the closure of GRP94 to trap the client protein (Huang et al., 2022). However, there is a lack of information on which co-chaperones might act with ENPL-1/GRP94 for proinsulin handling and processing. Our studies in *C. elegans* identified ASNA-1 as a new interactor of ENPL-1 and provided evidence consistent with the notion that ASNA-1 might be a co-chaperone of ENPL-1.

Both ASNA1 and GRP94 act to positively regulate insulin secretion in mice (Kim et al., 2018; Norlin et al., 2018). These studies showed that both proteins were essential for pancreas development and function, and specific knockdown in the pancreas led to a diabetic phenotype (Kim et al., 2018; Norlin et al., 2016, 2018). Our studies in *C. elegans* also showed that both proteins, which are well conserved with their mammalian counterparts, are positive regulators of insulin signaling. Notably, human ASNA1 can substitute for worm ASNA-1 for both growth and cisplatin detoxification functions (Hemmingsson et al., 2010; Kao et al., 2007), indicating conservation of function and that results obtained with worm ASNA-1 can be relevant for human biology. Knockdown of either of these two worm genes resulted in growth arrest and a severe defect in DAF-28/ILP secretion (Kao et al., 2007; Podraza-Farhanieh et al., 2020). We have previously shown that ENPL-1 binds DAF-28, and in the absence of *enpl-1*, DAF-28 pro-peptide levels are downregulated and that DAF-28 is found only in an unprocessed form. This very likely contributes to the defect in insulin secretion. However, very little is known at the subcellular level about how ASNA-1 regulates DAF-28/ILP secretion in *C. elegans*. Our analysis here indicates that ASNA-1 expressed specifically in neurons is responsible for insulin secretion in *C. elegans*, as the neuron-specific ASNA-1 depletion resulted in insulin secretion defect. These data, together with our demonstration of the interaction between ASNA-1 and ENPL-1 in DAF-28-expressing neurons supports the notion that ASNA-1 is required by ENPL-1 to maintain DAF-28 secretion. We note further that our previous work has shown that ENPL-1 has functions outside the ER compartment (Podraza-Farhanieh et al., 2020). This provides possible subcellular locations for the interaction between the two proteins. Roles outside the ER are not only a feature of worm ENPL-1. Rather, there is substantial evidence for such non-ER roles for mammalian GRP94/HSP90B1 (Frasson et al., 2009; Patel et al., 2013). Furthermore, it has been also shown that yeast and mammalian homologs of ASNA-1 are required for maintaining proper retrograde transport and retrieval of proteins with the HDEL motif from the Golgi to the ER (Norlin et al., 2016; Schuldiner et al., 2005). We note that ENPL-1 also has a C-terminal HSEL motif, which is required for its relocation to the ER (Hirate and Okamoto, 2006). Our global proteomic analysis reported Golgi and ER trafficking, and retrograde transport changes in the *asna-1* mutants as one of the main pathways affected by the loss of ASNA-1.

How might ASNA-1 and GRP94 act together to promote DAF-28 secretion? Our findings support the model that ENPL-1 binds to DAF-28 in the ER and transports it to sites in the Golgi where the dense core vesicles bud off. After releasing DAF-28 (via an unknown mechanism), unliganded ENPL-1 interacts with the oxidized chaperone form of ASNA-1 at the Golgi and is taken back to the ER by retrograde transport. ENPL-1, which is returned to the ER will be available to bind and transport new molecules of DAF-28. Increasing ENPL-1 levels by overexpression could lead to higher levels of the ENPL-1/DAF-28 pro-peptide complex formation that, if sufficient, could result in more DAF-28 being delivered to the dense core vesicles and consequently to some secretion and uptake by coelomocytes being detected. Only a partial bypass of the requirement of ASNA-1 by overexpression of ENPL-1 was detected. This observation would be consistent with a high initial level of ENPL-1 (upon overexpression) that would still require ASNA-1 to execute the Golgi to ER trafficking step. The greatly reduced binding between the ENPL-1 and ASNA-1 in *daf-28* mutants would likely be the result of reduced ER to Golgi transport of ENPL-1 in the absence of its DAF-28 client protein, thus reducing the requirement for ASNA-1 to retrieve it from the Golgi.

Our results show that interaction between ASNA-1 and ENPL-1 is DAF-28 dependent because in the *daf-28* mutants we observed a significant reduction in the interaction. Our previous studies have shown that ENPL-1 interacts with DAF-28 pro-peptide and that, in the absence of *enpl-1*, DAF-28 pro-peptide cannot be properly cleaved (Podraza-Farhanieh et al., 2020). These data and the fact that both proteins are essential for insulin secretion indicates that the interaction is related to insulin binding and possibly to processing. Although the genome of *C. elegans* encodes 40 insulin-like proteins (Pierce et al., 2001), we have chosen to study two ILPs, *daf-28* and *ins-4*, and their impact on interaction as it has been shown that, during maturation, these two ILPs are cleaved by two different proprotein convertases: DAF-28 is cleaved by KPC-1 prohormone convertase, whereas INS-4 is cleaved by EGL-3 prohormone convertase (Hung et al., 2014). Our previous work indicated that DAF-28 pro-peptide is not cleaved in *kpc-1* mutants, leaving DAF-28 in the unprocessed form (Podraza-Farhanieh et al., 2020), which is consistent with earlier findings (Hung et al., 2014). Interestingly, the absence of these two ILPs had different impacts on the interaction between ASNA-1 and ENPL-1. First, we observed increased interaction in the *kpc-1(gk8)* mutants, indicating that the DAF-28 pro-peptide is required for the binding between ASNA-1 and ENPL-1. Second, we found that, in the absence of *ins-4*, the interaction level increases possibly via an increase in DAF-28 levels.

These contrasting results might indicate that the interaction between ASNA-1 and ENPL-1 is directed towards a specific ILP, DAF-28, and not towards all 40 ILPs expressed by *C. elegans*. It remains likely that other worm ILPs, such as INS-2, that require KPC-1 prohormone convertase for their processing might also require the ENPL-1/ASNA-1 complex for their function (Hung et al., 2014). We do not rule out the possibility that client proteins that do not need KPC-1 prohormone convertase for their function might use the ASNA-1/ENPL-1 complex. The *unc-31* mutant was used in the co-immunoprecipitation studies because *unc-31* mutants accumulate increased numbers of mature DCVs compared with wild-type that are not primed for docking (Hammarlund et al., 2008). These mature DCVs presumably contain fully processed DAF-28 and correspondingly the level of DAF-28 pro-peptide (found in immature DCVs) is likely to be lower. However, the secretion of many neuropeptides is perturbed in *unc-31/CAPS* mutants and the possibility remains that defects in the secretion of some other neuropeptide might affect that binding between the two proteins (Cai et al., 2009; Hammarlund et al., 2008; Speese et al., 2007).

ASNA-1 has been mainly described as a protein that promotes the insertion of tail-anchored proteins into the ER (Favaloro et al., 2008; Schuldiner et al., 2008; Stefanovic and Hegde, 2007). The only known interactors of ASNA-1 or its homologs are proteins that take part in TAP insertion (Vilardi et al., 2011). However, it has been shown that ASNA-1 and its yeast homolog, Get3, can act as a holdase chaperone that under oxidative stress conditions protects proteins from oxidative stress damage (Mateja et al., 2009; Powis et al., 2012; Raj et al., 2021; Voth et al., 2014). Those changes involve structural rearrangements of the protein, including disulfide bond formation, zinc release and oligomerization (Voth et al., 2014). Our previous study showed that *C. elegans* ASNA-1 is normally present both in the oxidized and reduced state in the animal. The oxidized state is favored under the high ROS conditions (Raj et al., 2021). Forcing ASNA-1 to adopt the oxidized state impairs TAP insertion but does not affect insulin secretion. This indicates that these two functions can be separated, and that oxidized ASNA-1 is responsible for the role in insulin secretion (Raj et al., 2021). Our current results show that the interaction between ASNA-1 and ENPL-1 is increased under high ROS conditions, which leads to the oxidation of ASNA-1 and to impaired TAP insertion function. This allows us to propose that the oxidized form of ASNA-1 is most likely the binding partner of ENPL-1. More broadly, GRP94 has diverse roles that have an impact on human health. The discovery that ASNA-1 is functionally linked to it can be a starting point for genetic or drug-based interventions that modulate its activity.

Insulin secretion is central to organism metabolism and a healthy life. Much is known about how insulin secretion can be stimulated (e.g. by glucose) but considerably less is known about insulin maturation. Our study links ENPL-1 and ASNA-1, and provides information on how they might cooperate to produce insulin maturation and secretion, and prevent diabetes.

## MATERIALS AND METHODS

### *C. elegans* genetics and maintenance

Animals were maintained under standard conditions at 20°C on nematode growth media (NGM) plates (Brenner, 1974). N2 is the wild-type parent for all the strains in the study. The *daf-28::mNeonGreen (PHX3050*) strain was created by SunyBiotech using the CRISPR/Cas9 technique by inserting a flexible linker and mNeonGreen before the stop codon of *daf-28*. The multi-copy ASNA-1::GFP(*svIs56*) transgenic animals, the multi-copy DAF-28::GFP(*svIs69*) transgenic animals and *asna-1(ok938)* mutants, which were maintained *in trans* to the hT2(*qIs48*) balancer, have been previously described (Kao et al., 2007). The single-copy 3xFlag::ENPL-1(*knuSi222*), the single copy 3xFlag:: ENPL-1(ΔCBD) *knuSi430* with an in-frame deletion of the Client binding domain, the knock-in strains ENPL-1::mKATE2::enpl-1(*PHX698*) and the double-tagged ollas::DAF-28::Myc(*rawEx11*) have been previously described (Podraza-Farhanieh et al., 2020). The ASNA-1::mNeonGreen::AID [*PHX2249*] was created by CRISPR/Cas9 knock-in of two tags, mNeonGreen and AID, at the C terminus of the protein of ASNA-1 by Sunybiotech. *enpl-1(ok1964)* was outcrossed eight times and maintained *in trans* to the nT1(qIs51) balancer. *sod-2(gk257)*, *daf-28(tm2308)*, *ins-4(tm3620)*, *kpc-1(gk8)*, *unc-31(e928)* and *otIs356*-expressing nuclear-localized pan neural tag::RFP and *oyIs84* were obtained from the *Caenorhabditis* Genetics Center (University of Minnesota, Minneapolis, USA) (Table S2).

### AMAN-2::wrmScarlet construction and transgene generation

The wrmscarlet gene was inserted by Gibson ligation between the 2.4 kb osm-6 promoter and the 3′UTR of tbb-2 to generate pGK234. The plasmid pZCS16 was the source of wrmscarlet. pGK234 was used as a starting point to insert the 342nt aman-2 coding fragment amplified from wild-type DNA upstream of and in frame with wrmscarlet to yield pGK235. The plasmid was injected at 10 ng/μl along with pCC::RFP (50 ng/μl) to generate the transgenic line containing rawEx90. The transgene-bearing worms were backcrossed to wild type three times before use.

### Antibodies and western blotting

Wild-type animals or indicated mutants were directly washed using M9 from the plates. Animals were lysed in Next Advance Bullet Blender Homogenizer in buffer containing 10 mM Tris-HCl (pH 7.4), 150 mM NaCl, 5 mM EDTA and 0.5%NP40 (between 80 µl and 200 µl) using 0.2 mm stainless steel beads for 3 min at 4°C, followed by centrifugation at 18,400 ***g*** for 20 min at 4°C. Protein estimation was conducted using a BCA assay. Reducing SDS-PAGE was performed as described previously (Raj et al., 2021). Proteins were separated by SDS-PAGE and blotted onto nitrocellulose membranes. Proteins were detected using the following antibodies: anti-ASNA-1 (Kao et al., 2007), anti-GFP (3H9, ChromoTek, RRID: AB_10773374, 3h9-100, LOT 80626001AB, 1:1000), anti-GRP94 (9G10, Thermo Fisher Scientific, MA3-016; RRID: AB_2248666; 1:1000), anti-Flag (M2, Sigma-Aldrich, RRID: AB_262044, F1804, LOT SLCD3990, 1:1000), anti-tubulin (Sigma-Aldrich, RRID: AB_477579, T5168, LOT 00000089494, 1:5000) and anti-OLLAS (L2, Novus Biologicals, RRID: AB_1625980, NBP1-06713SS, 1:1000). The secondary antibodies, used at a dilution of 1:5000, were HRP conjugated: goat anti-rat (GE Healthcare Life Sciences, RRID: AB_772207, NA935, LOT 16918042), sheep anti-mouse (GE Healthcare Life Sciences, RRID: AB_772193, NA9310, LOT 16921365) and donkey-anti-rabbit (GE Healthcare Life Sciences, RRID: AB_2722659, NA934, LOT 9670531). Supersignal West Femto detection reagent (Thermo Fisher Scientific) was used to generate a signal, which was detected using a LAS1000 machine (Fujifilm).

### Co-immunoprecipitation

Worms were grown on the NGM plates for 4 days at 20°C and lysed as described above. Total protein lysates (800-1000 µg) were then added to 25 μl of anti-GFP magnetic beads (ChromoTek GFP-Trap) and tumbled end-over-end for 1 h at 4°C. Beads were separated with a magnet and washed three times for 10 min with 600 μl of wash buffer [10 mM Tris-HCl (pH 7.4), 150 mM NaCl and 5 mM EDTA]. Proteins were eluted by re-suspending the washed beads in 20 μl of 2× loading dye with β-mercaptoethanol, followed by heating for 10 min at 95°C. SDS PAGE was performed as described above.

### Neuropeptide secretion assays

One-day-old adult animals were anaesthetized using 10 mM levamisole, mounted on 2% agarose pads and directly imaged. DAF-28::GFP uptake into coelomocytes of adult worms was measured directly after preparing the slide. Control samples and indicated mutants were measured using the same settings in parallel.

### H_2_O_2_ exposure

Hermaphrodites were grown on the NGM plates for 4 days. After the indicated time, animals were washed from the NGM plates and incubated with 5 mM H_2_O_2_ for 30 min. Worms were harvested and processed for lysate preparation and co-immunoprecipitation.

### Auxin-mediated depletion

Water-soluble auxin-containing [naphthaleneacetic acid (K-NAA), PhytoTech, LOT HKA0610009, 15165-79-4] plates were prepared on the day of use by adding the indicated concentration of auxin to NGM plates after cooling the agar down to 56°C. The plates containing auxin were kept in darkness during preparation and experimental procedures.

#### Neuronal depletion

*ASNA-1::mNeonGreen::AID;reSi7* animals were grown to L1-L2 stage. Staged animals were placed on 2 mM auxin plates for 48 h and kept in the darkness. After the indicated time, fluorescence microscopy and DIC pictures were taken.

#### Somatic depletion

*ASNA-1::mNeonGreen::AID; ieSi57* animals were grown to L4 stage. L4 animals were placed on 1 mM auxin plates for 48 h and kept in the darkness. After 48 h, mothers were taken from the plate. After another 24 h, the laid progeny/unhatched eggs were counted.

### Confocal microscopy

One-day-old adult animals were anaesthetized using 10 mM levamisole, mounted on 2% agarose pads and directly imaged. The fluorescence signal was analyzed at 488 nm and 561 nm using a confocal laser scanning microscope (LSM880, Carl Zeiss) with C-Apochromat 40×/1.2 water immersion objective lens. Image processing was performed using ZEN Lite (Carl Zeiss) software.

### RNA extraction and quantitative RT-PCR (qPCR)

Worms were grown on the NGM plates for 4 days at 20°C. Worms were synchronized by allowing a mixed-stage worm suspension in M9 buffer to settle for 3 min and collecting the supernatant, which contained embryos and L1 larva. These were placed on fresh NGM plates for 48 h to grow until the young adult stage. Worms were re-suspended in 75 µl Nucleozol (Macherey-Nagel). After lysis by three rounds of freeze/thaw (37°C and ethanol/dry ice), the RNA was extracted using the Aurum Total RNA Mini Kit (Bio-Rad). cDNA was synthesized using the iScript cDNA Synthesis Kit (Bio-Rad). qPCR was performed on a StepOnePlus Real-Time PCR System (Applied Biosystems) instrument using KAPA SYBR FAST qPCR Kit (Kapa Biosystems). The comparative Ct method was used to analyze the results and the reference gene used for the analysis was *CDC-42.*

### Proteomic analysis

Staged, adult N2 and *asna-1(ok938)* animals were collected and lysed in 2% SDS in 50 mM of triethylammonium bicarbonate (TEAB) buffer. Total protein estimation was estimated using BCA estimation. Four representative reference pools were made by taking equal protein amounts from wild-type and *asna-1(ok938)* mutant samples and combining into a wild-type, a mutant and a wild-type/mutant reference.

Samples were subjected to relative quantitative mass spectrometry using TMT performed by The Proteomics Core Facility at Gothenburg University and analyzed by The Bioinformatics Core Facility at Gothenburg University.

### Detailed description of mass spectroscopy analysis

#### Protein extraction, digestion and tandem mass tag (TMT) labeling

Worms were lysed in buffer containing 2%SDS in 50 mM TEAB. Protein concentration of the lysates was determined using Pierce BCA Protein Assay Kit (Thermo Fisher Scientific). The samples were digested with trypsin using the filter-aided sample preparation (FASP) method (Wiśniewski et al., 2009). Briefly, samples (30 µg) were reduced with 100 mM dithiothreitol (60°C, 30 min) transferred to 10 kDa MWCO Pall Nanosep centrifugation filters (Sigma-Aldrich), washed repeatedly with 8 M urea and once with digestion buffer [DB, 1% sodium deoxycholate (SDC) and 50 mM TEAB] before alkylation with 10 mM methyl methanethiosulfonate in DB (30 min, room temperature). Digestion was performed by the addition of Pierce MS grade trypsin (0.3 µg, at 37°C, Thermo Fisher Scientific) and incubated overnight. An additional dose of trypsin (0.3 µg) was added and incubated for another 3 h. Peptides were collected by centrifugation and labelled using TMT 10-plex isobaric mass tagging reagents (Thermo Fisher Scientific) according to the manufacturer's instructions. The peptide samples were combined and SDC was removed by acidification with 10% TFA. The samples were further purified using HiPPR Detergent Removal Resin (Thermo Fisher Scientific) according to the manufacturer's instructions. The TMT-set was pre-fractionated into 40 fractions by basic reverse-phase liquid chromatography (bRP-LC) using a Dionex Ultimate 3000 UPLC system (Thermo Fisher Scientific). Peptide separation was performed using a reverse-phase XBridge BEH C18 column (3.5 μm, 3.0×150 mm, Waters) and in a gradient from 3% to 90% acetonitrile in 10 mM ammonium formate buffer (pH 10.00) over 35 min. The fractions were concatenated into 20 fractions, dried and reconstituted in 3% acetonitrile and 0.2% formic acid.

#### LC-MS/MS analysis

The fractions were analyzed on an Orbitrap Fusion Tribrid mass spectrometer interfaced with an Easy-nLC1200 liquid chromatography system (both Thermo Fisher Scientific). Peptides were trapped on an Acclaim Pepmap 100 C18 trap column (100 μm×2 cm, particle size 5 μm, Thermo Fisher Scientific) and separated on an in-house packed analytical column (75 μm×35 cm, particle size 3 μm, Reprosil-Pur C18, Dr Maisch) using a gradient from 3% to 80% acetonitrile in 0.2% formic acid over 80 min at a flow of 300 nl/min. MS scans were performed at a resolution of 120,000, m/z range 380-1380. The most intense multiply charged precursor ions were selected for MS2 fragmentation with top speed cycle of 3, 0.7 m/z isolation window and a dynamic exclusion within 10 ppm for 60 s. Produced MS2 fragment ions were detected in the ion trap followed by multinotch (simultaneous) isolation of the top five most abundant fragment ions for further fragmentation (MS3) by higher-energy collision dissociation (HCD) at 65% and detection in the Orbitrap at 50,000 resolutions, m/z range 100-500.

#### Proteomic data analysis

Identification and relative quantification were performed using Proteome Discoverer version 2.2 (Thermo Fisher Scientific). The search was performed by matching against *Caenorhabditis elegans* Uniprot Database (November 2018) using Mascot 2.5.1 (Matrix Science) with a precursor mass tolerance of 5 ppm and fragment mass tolerance of 0.6 Da. Tryptic peptides were accepted with zero missed cleavage; methionine oxidation was set as a variable modification; cysteine alkylation, TMT-modification on lysine and peptide N-termini were set as fixed modifications. A percolator was used for the validation of identified proteins. TMT reporter ions were identified in the MS3 HCD spectra with 3 mmu mass tolerance, and the TMT reporter intensity values for each sample were normalized on the total peptide amount. Only peptides unique to a given protein were considered for protein quantification. The mass spectrometry proteomics data have been deposited in the ProteomeXchange Consortium via the PRIDE partner repository with the dataset identifier PXD038504.

### Statistical analysis

Statistical analysis was performed using Prism 9 software (GraphPad Software). Statistical significance was determined using a two-tailed, unpaired Student's *t*-test. *P*<0.05 indicated statistical significance (*P<0.05, **P<0.01, ***P<0.001, ****P<0.0001).

## Supplementary Material

10.1242/develop.201035_sup1 Supplementary information
