# Supplementary material for: A proinsulin-dependent interaction between ENPL-1 and ASNA-1 in neurons is required to maintain insulin secretion in C. elegans
Source: Development. 2023 Mar 20;150(6):dev201035. doi: 10.1242/dev.201035 (PMC10112894; doi:10.1242/dev.201035)
Supplement: Supplementary information [file develop-150-201035-s1.pdf]

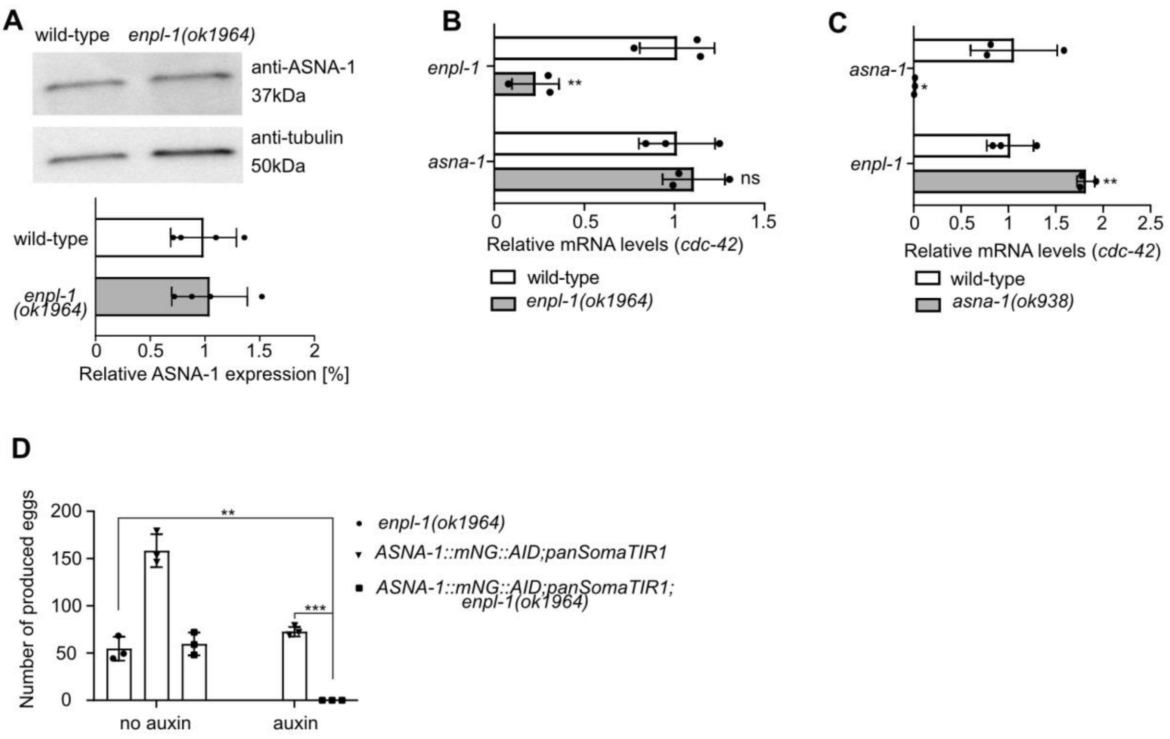

Fig. S1. (A) Western blot analysis of lysates prepared from adult wild-type and *enpl-1(ok1964)* mutants. The membrane was probed with an anti-ASNA-1 antibody. Anti- tubulin antibody was used to determine equal loading. Quantification shows relative ASNA-1 expression in indicated strains. (B) Relative qPCR analysis of *enpl-1* and *asna-1* expression in adult wild-type and *enpl-1 (ok1964)* mutants. Statistical significance was determined using the two-tailed t-test (**P<0.01). Bars represent mean ± SD. *cdc42* was used as a normalizing control for the experiment. The experiment was performed in triplicate. (C) Relative qPCR analysis of *asna-1* and *enpl-1* expression in adult wild-type and *asna- 1(ok938)* mutants. Statistical significance was determined using the two-tailed t-test (*P<0.05**, P<0.01). Bars represent mean ± SD. *cdc42* was used as a normalizing control for the experiment. The experiment was performed in triplicate. (D) Number of produced eggs by the indicated strains without auxin and exposed to 1mM of auxin for 72h. The experiment was performed in triplicate. Statistical significance was determined using the two-tailed t-test (**P<0.01, ***P<0.001). Bars represent mean ± SD.

Development • Supplementary information

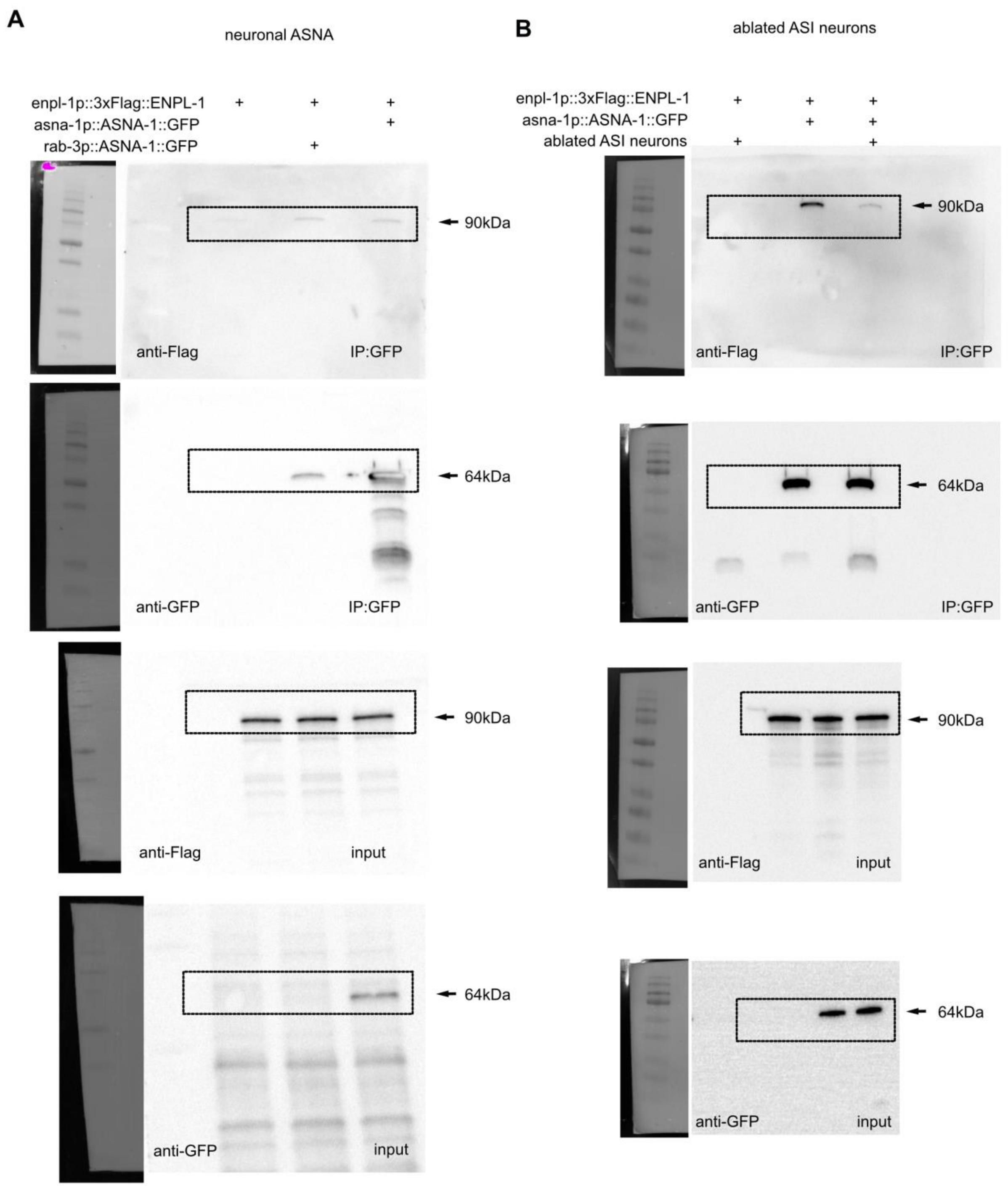

Fig. S2. (A) Full uncropped images of Western Blots representing co-immunoprecipitation experiments with anti-GFP affinity beads from lysates of adult animals expressing 3xFlag::ENPL-1, co-expressing 3xFlag::ENPL-1 + rab3p::ASNA-1::GFP, and 3xFlag::ENPL-1 + asna-1p::ASNA-1::GFP. The membranes were probed with anti-GFP and anti-Flag antibodies. (B) Full uncropped images of Western Blots representing co-immunoprecipitation experiments with anti-GFP affinity beads from lysates of adult animals expressing 3xFlag::ENPL-1, co-expressing 3xFlag::ENPL-1 + ASNA-1::GFP with and without *oyIs84* followed by western blot analysis. The membranes were probed with anti-GFP and anti-Flag antibodies.

Development • Supplementary information

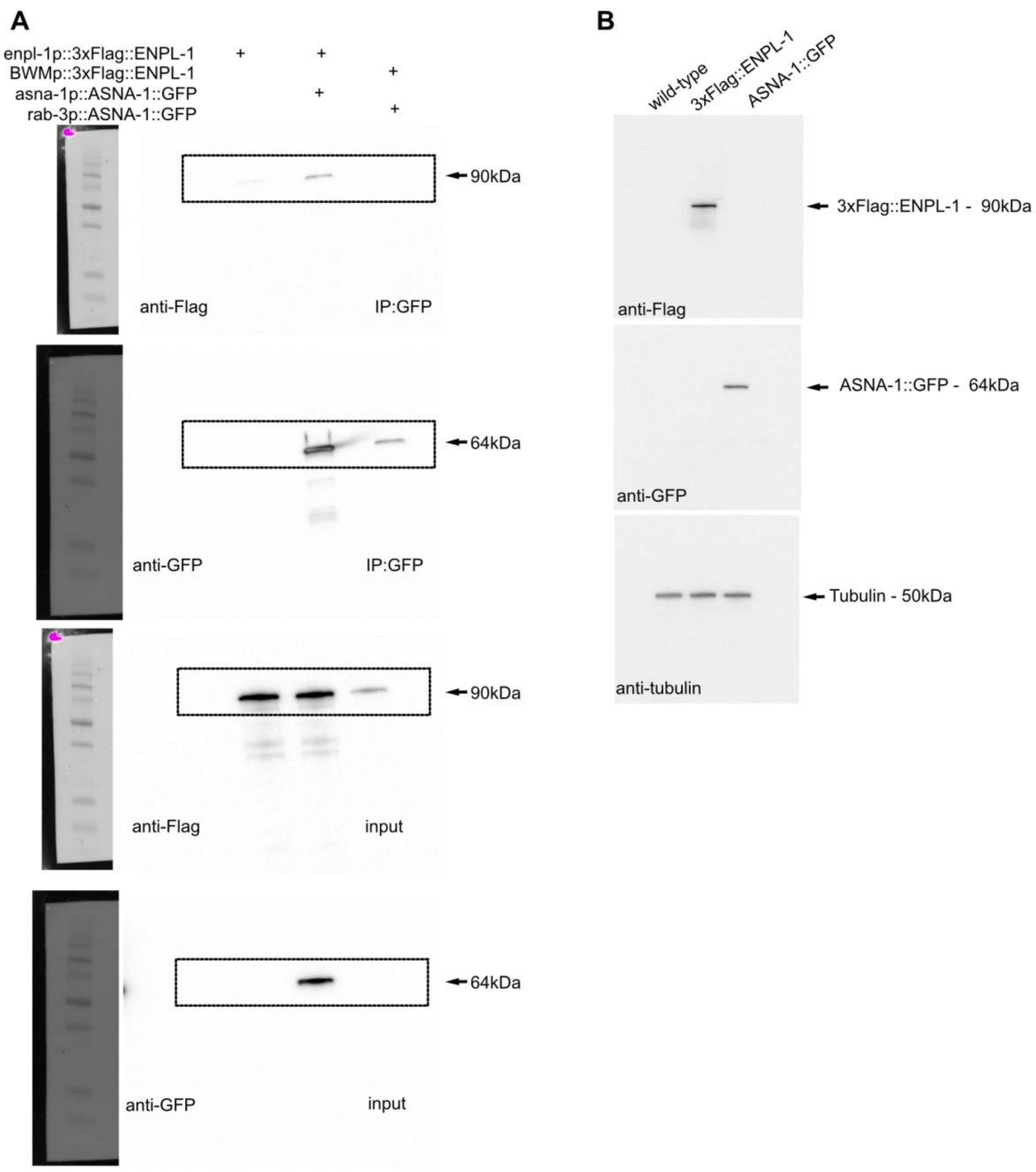

Fig. S3. (A) Full uncropped images of Western Blots representing co-immunoprecipitation experiments using anti-GFP affinity beads from lysates of adult animals expressing 3xFlag::ENPL-1 under the control of the *enpl-1* promoter or the body wall muscle promoter (myo-3p) and ASNA-1::GFP under control of the *asna-1* promoter and neuronal promoter (rab3p) followed by western blot analysis. The membranes were probed with anti-GFP and anti-Flag antibodies. (B) Full uncropped images of Western Blots representing the specificity of anti-GFP and anti-Flag antibodies. Lysates from wild-type, 3xFlag::ENPL-1 and ASNA-1::GFP were used. The membranes were probed with anti-GFP, anti-Flag and anti-tubulin antibodies.

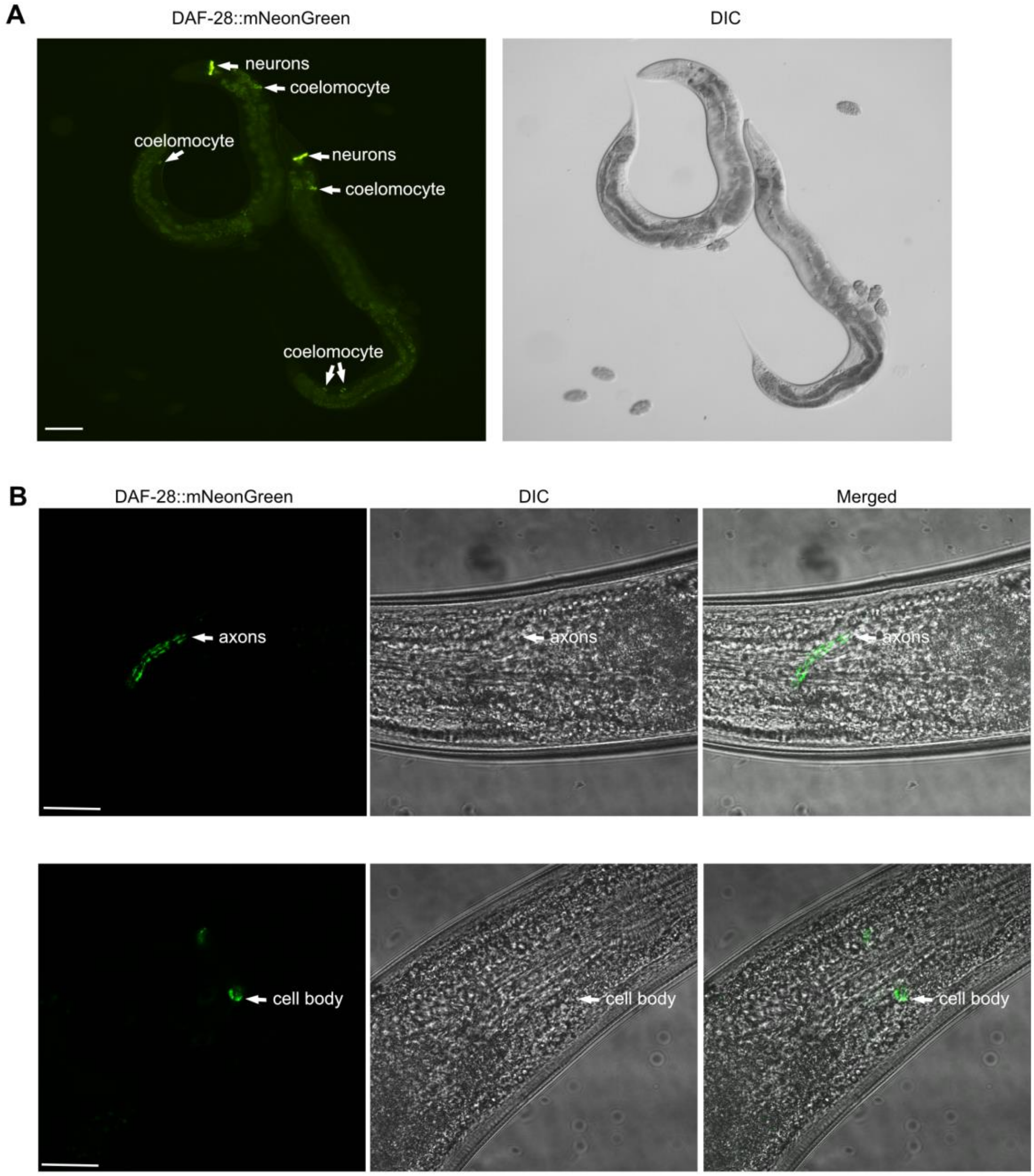

Fig. S4. Expression of DAF-28::mNeonGreen. (A) Representative fluorescence and differential interference contrast (DIC) images of adult animals expressing DAF-28::mNeonGreen (*syb3050*) at 10X magnification. White arrows indicate the structures where DAF-28::mNeonGreen is expressed. Scale bar: 100 µm. (B) Representative confocal images of adult animals expressing DAF-28::mNeonGreen at 63X magnification. White arrows indicate the structures where DAF-28::mNeonGreen is expressed. Scale bar: 20 µm.

Development • Supplementary information

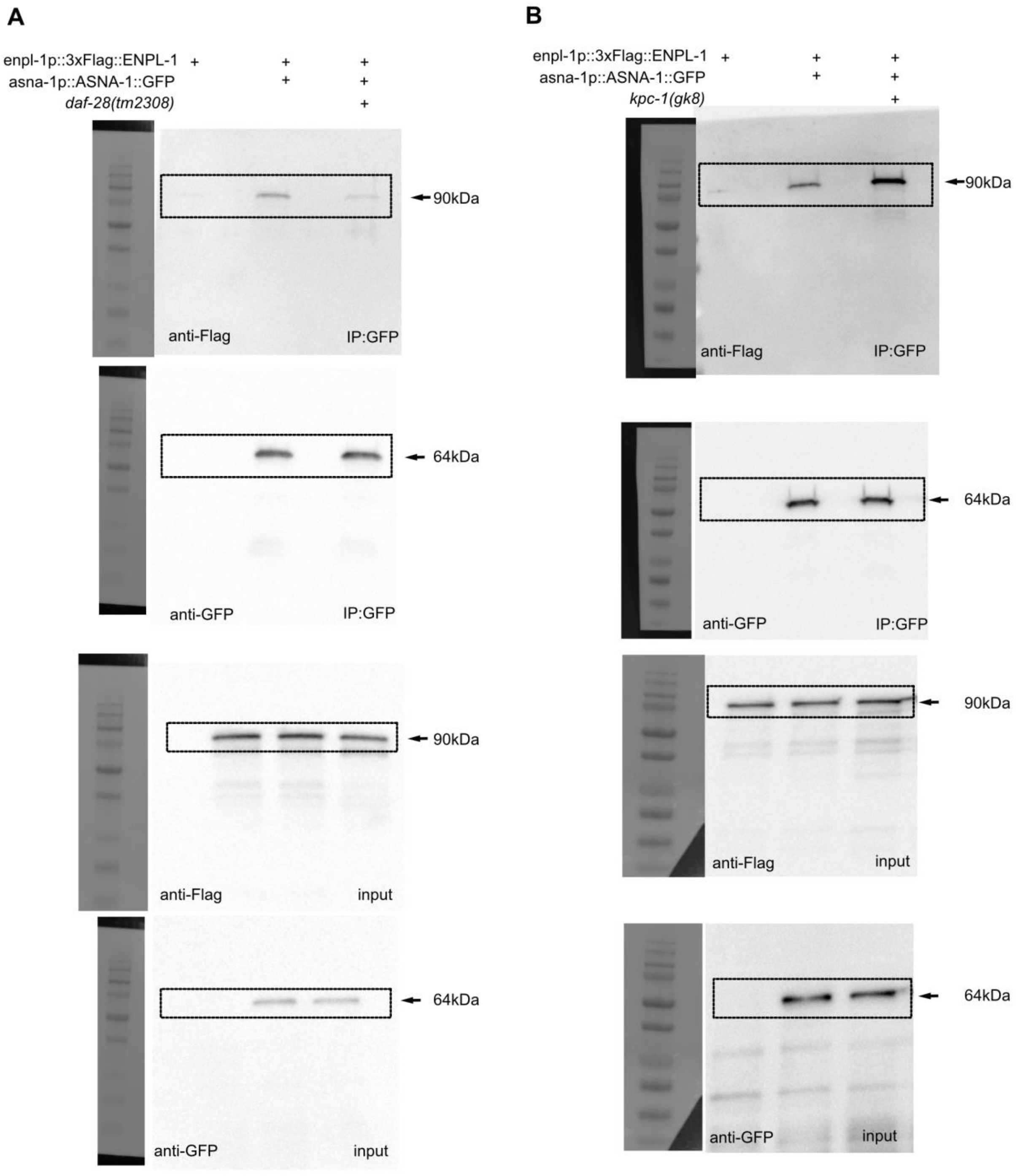

Fig. S5. (**A**) Full uncropped images of Western Blots representing co-immunoprecipitation experiments with anti-GFP affinity beads from lysates of adult animals expressing 3xFlag::ENPL-1 or co-expressing 3xFlag::ENPL-1 + ASNA-1::GFP in *daf-28(tm2308)* mutants. The membranes were probed with anti-GFP and anti-Flag antibodies. (B) Full uncropped images of Western Blots representing co-immunoprecipitation experiments with anti-GFP affinity beads from lysates of adult animals expressing 3xFlag::ENPL-1 or co-expressing 3xFlag::ENPL-1 + ASNA-1::GFP in *kpc-1(gk8)* mutants. The membranes were probed with anti-GFP and anti-Flag antibodies.

Development • Supplementary information

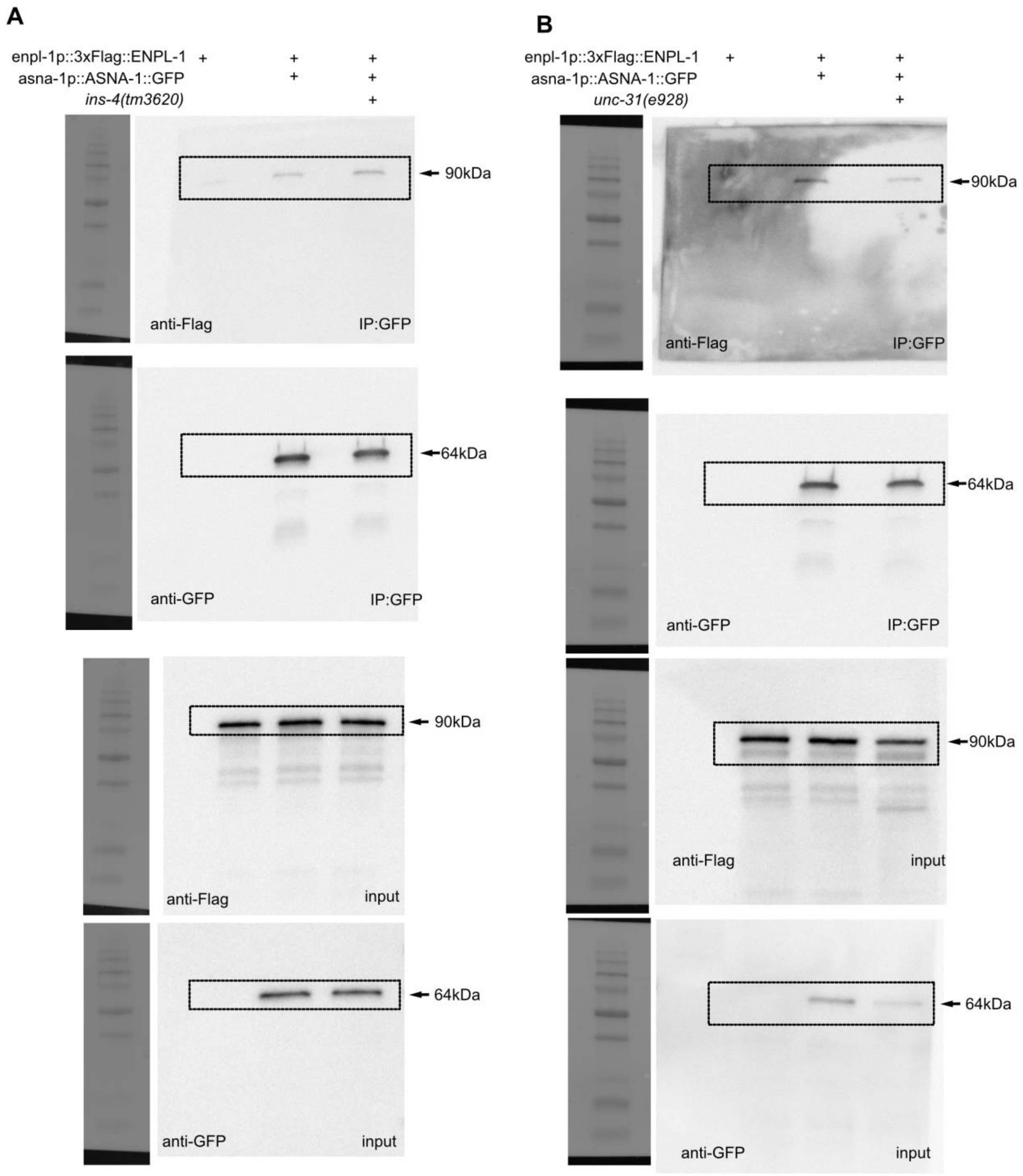

Fig. S6. (**A**) Full uncropped images of Western Blots representing co-immunoprecipitation experiments with anti-GFP affinity beads from lysates of adult animals expressing 3xFlag::ENPL-1 or co-expressing 3xFlag::ENPL-1 + ASNA-1::GFP in *ins-4(tm3620)* mutants. The membranes were probed with anti-GFP and anti-Flag antibodies. (B) Full uncropped images of Western Blots representing co-immunoprecipitation experiments with anti-GFP affinity beads from lysates of adult animals expressing 3xFlag::ENPL-1 or co-expressing 3xFlag::ENPL-1 + ASNA-1::GFP in *unc- 31(e928)* mutants. The membranes were probed with anti-GFP and anti-Flag antibodies.

Development • Supplementary information

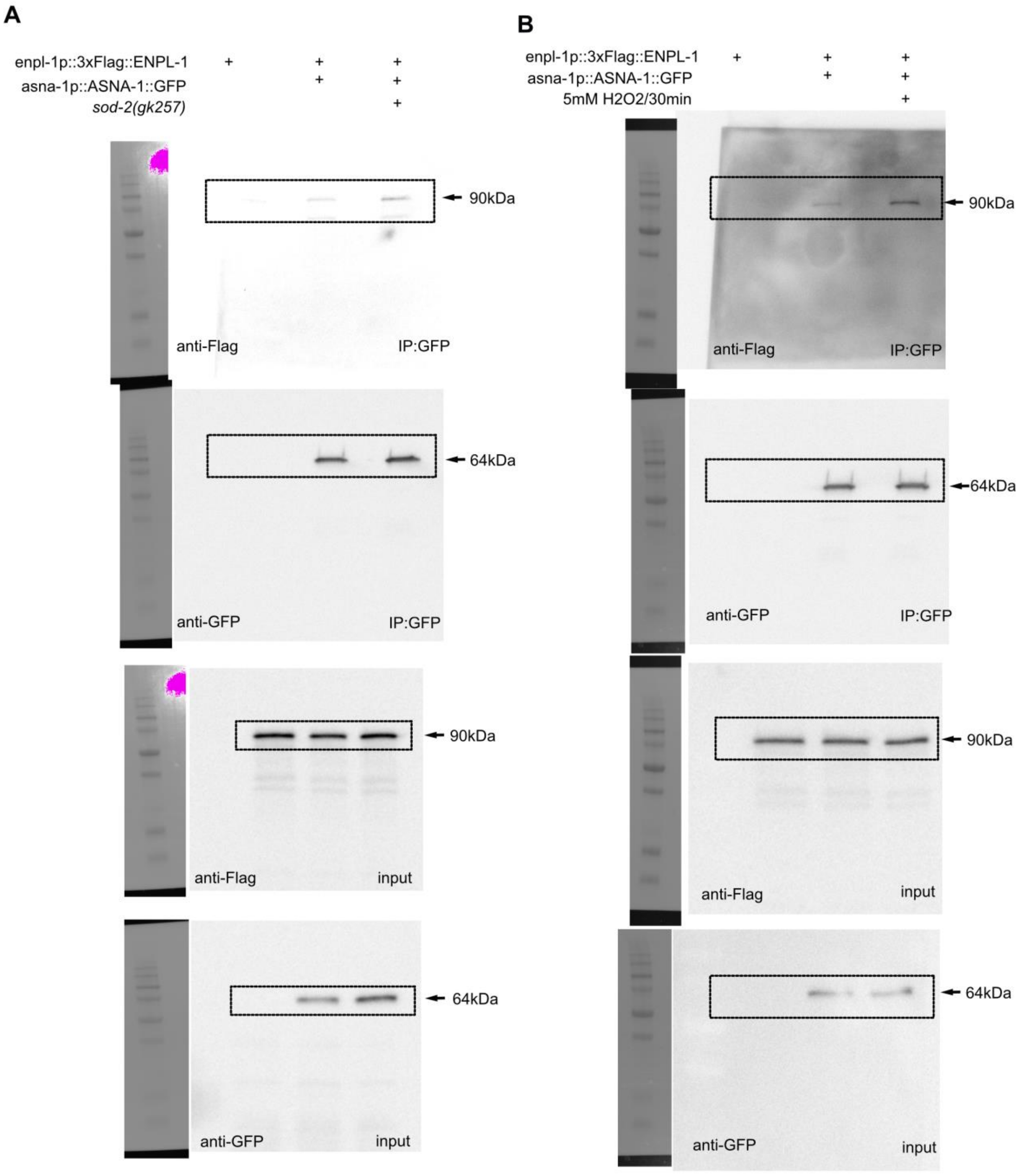

Fig. S7. (**A**) Full uncropped images of Western Blots representing co-immunoprecipitation experiments with anti-GFP affinity beads from lysates of adult animals expressing 3xFlag::ENPL-1 or co-expressing 3xFlag::ENPL-1 + ASNA-1::GFP in *sod-2(gk257)* mutants. The membranes were probed with anti-GFP and anti-Flag antibodies. (B) Full uncropped images of Western Blots representing co-immunoprecipitation experiments with anti-GFP affinity beads from lysates of adult animals expressing 3xFlag::ENPL-1 or co-expressing 3xFlag::ENPL-1 + ASNA-1::GFP exposed to 5mM H_2O_2 for 30min. The membranes were probed with anti-GFP and anti-Flag antibodies.

Development • Supplementary information

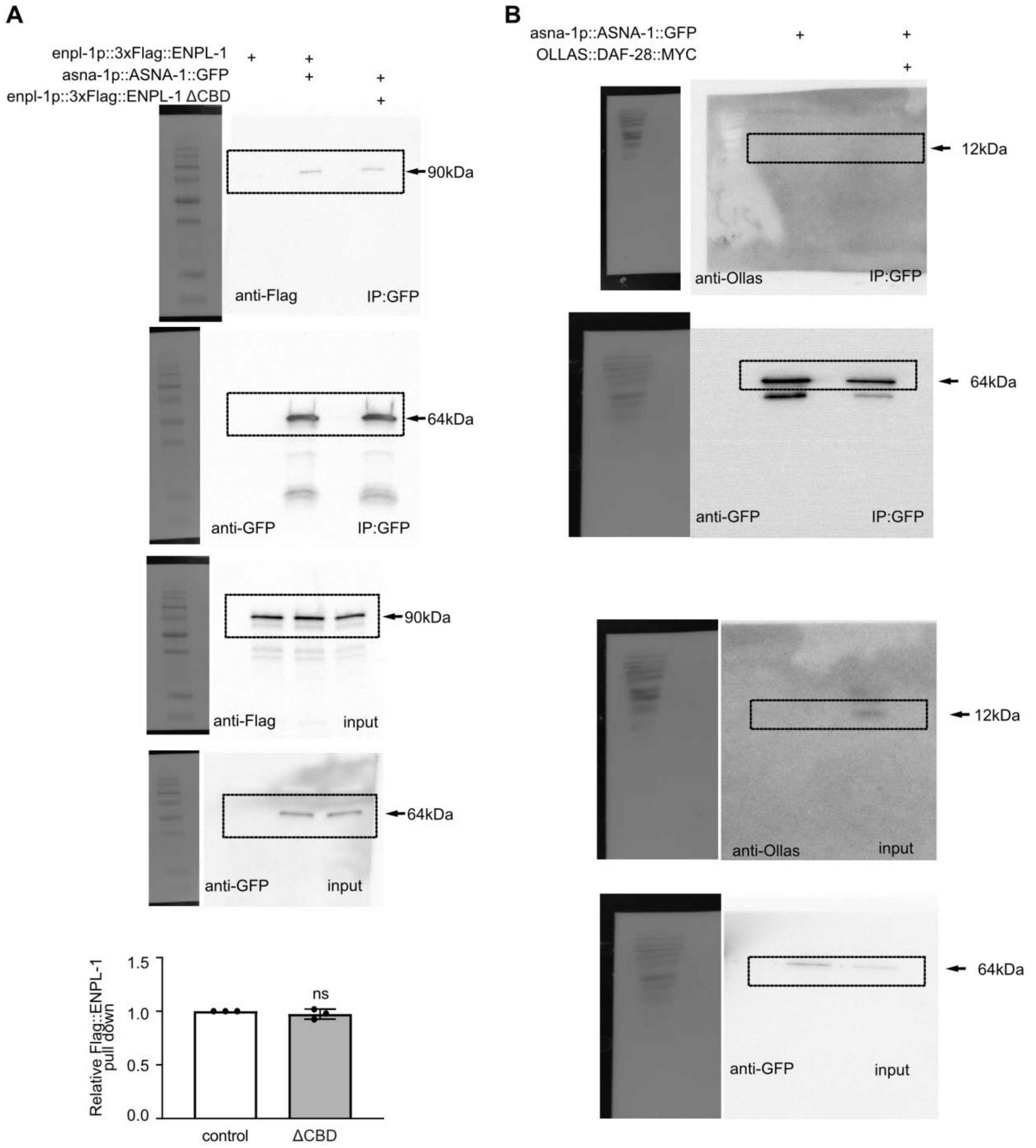

Fig. S8. (**A**) Full uncropped images of Western Blots representing co-immunoprecipitation experiments using anti-GFP affinity beads from lysates of adult animals expressing 3xFlag::ENPL-1, co-expressing 3xFlag::ENPL-1 + ASNA-1::GFP or co-expressing 3xFlag::ENPL-1(ΔCBD) + ASNA- 1::GFP. The membranes were probed with anti-GFP and anti-Flag antibodies. The experiments were performed in triplicate. Quantification shows relative levels of 3xFlag::ENPL-1 immunoprecipitation in the indicated strains. Statistical significance was determined using the two-tailed t-test. Bars represent mean ± SD. (B) Full uncropped images of Western Blots representing co-immunoprecipitation experiments with anti-GFP affinity beads from lysates of adult animals expressing ASNA-1::GFP with and without OLLAS::DAF-28::MYC. The membranes were probed with anti-Ollas and anti-GFP antibodies.

Development • Supplementary information

Table S1. Global proteomic analysis of *asna-1(ok938)* mutants compared to wild-type (N2). The total number of detected proteins in this analysis with Accession, Description, # Peptides, #PSMs, # Unique Peptides, MW [kDa] and Abundances (Sheet 1). Differentially expressed proteins in *asna-1(ok938)* mutants with fold change unlogged, pValue and FDR 1% (Sheet 2). List of proteins in Reactome top enriched pathways (Sheet 3). List of top 20 up and down-regulated proteins (Sheet 4).

Click here to download Table S1

Table S2. *C. elegans* strain list used in this study.

Click here to download Table S2
