# Supplementary material for: A proinsulin-dependent interaction between ENPL-1 and ASNA-1 in neurons is required to maintain insulin secretion in C. elegans
Source: Development. 2023 Mar 20;150(6):dev201035. doi: 10.1242/dev.201035 (PMC10112894; doi:10.1242/dev.201035)
Supplement: Supplementary file 1 [file develop-150-201035_review_history.pdf]

A proinsulin dependent interaction between ENPL-1 and ASNA-1 in neurons is required to maintain insulin secretion in *C. elegans*

Agnieszka Podraza-Farhanieh, Dorota Raj, Gautam Kao and Peter Naredi
DOI: 10.1242/dev.201035

Editor: Swathi Arur

Review timeline
Original submission: 15 June 2022
Editorial decision: 25 July 2022
First revision received: 5 December 2022
Editorial decision: 3 January 2023
Second revision received: 10 February 2023
Accepted: 13 February 2023

Original submission

First decision letter

MS ID#: DEVELOP/2022/201035

MS TITLE: Proinsulin dependent interaction between ENPL-1/GRP94 and ASNA-1 in neurons is required to maintain insulin secretion in C. elegans.

AUTHORS: Agnieszka Podraza-Farhanieh, Dorota Raj, Gautam Kao, and Peter Naredi

I have now received all the referees' reports on the above manuscript, and have reached a decision. As you will see, the referees express considerable interest in your work, but provide recommendations to to address data rigor, biological significance of the phenomena observed as well as addressing whether ASNA-1 and ELP-1 are required *in vivo* for DAF-28 function. If you are able to revise the manuscript along the lines suggested, which involves further experiments, I will be happy receive a revised version of the manuscript. Your revised paper will be re-reviewed by the original referees. Please also note that Development will normally permit only one round of major revision.

Please attend to all of the reviewers' comments and ensure that you clearly highlight all changes made in the revised manuscript. Please avoid using 'Tracked changes' in Word files as these are lost in PDF conversion. I should be grateful if you would also provide a point-by-point response detailing how you have dealt with the points raised by the reviewers in the 'Response to Reviewers' box. If you do not agree with any of their criticisms or suggestions please explain clearly why this is so.

Reviewer 1

Advance summary and potential significance to field

Podraza-Farhanieh and colleagues report that a chaperone required for secretion of insulin-like peptides interacts with a ER factor that has been primarily studied in the context of protein translocation into the ER. The authors use a C. elegans model, which permits engineering of the loci that encode the chaperone ENPL-1, its interacting partner ASPN-1, and the insulin-like peptide DAF-28. The study integrates biochemical analysis of protein-protein interactions and phenotypic

© 2023. Published by The Company of Biologists under the terms of the Creative Commons Attribution License (https://creativecommons.org/licenses/by/4.0/).

analysis of C. elegans mutants to show that ASPN-1 is required for ILP release. The main points of the study - that ASPN-1 interacts with ENPL-1 and is required for ILP release - are supported by the data presented. These points, furthermore, advance understanding of molecular mechanisms that underlie ILP signaling and constitute significant discoveries.

Comments for the author

The manuscript has several weaknesses that should be addressed.

1. Throughout the manuscript, summary data are presented as bar graphs. These should be replaced with scatter plots that clearly show the distribution of individual measurements.

2. In several western blots there is a faint ENPL-1::FLAG signal under control conditions (anti-GFP pull-downs in the absence of ASPN-1::GFP). The authors should quantify ENPL-1::FLAG immunoreactivity to demonstrate the specificity of the ENPL-1::FLAG co-immunoprecipitation with ASPN-1::GFP

2. The cellular expression of ASNA-1 is initially reported as restricted to or enriched in ASI and ASJ, neurons that produce the ILP DAF-28. Micrographs of an asna-1 reporter allele, however, suggest a much broader expression pattern (Figure 2). Claims of specific expression should be tempered and the authors should clarify their description of which cells express ANSA-1.

3. The authors state that ASNA-1 and ENPL-1 co-localize in neurons but the expression shown in Figure 1B does not clearly show co-localization. The degree of co-localization should be measured (the R2 value included in the figure is not clearly explained) and an appropriate negative control should be used.

4. In Figure 2 the authors label a transgene that drives expression of a component of the auxin-induced degradation system as 'reSi7' - this will not mean anything to most readers and the authors should use a label that informs the reader about the function of the transgene.

5. The authors show that mutation of genes required for ILP processing and release affect the interaction between ASPN-1 and ENPL-1. They also test whether mutation of another ILP, INS-4, affects this interaction and find that in ins-4 mutants there is increased association of ASPN-1 and ENPL-1. The rationale for this experiment is not clear, nor is it clear why loss of INS-4 affects the ASPN-1/ENPL-1 interaction.

6. p. 7 line 14 - 'cleaved by the EGL-3' should be 'cleaved by the EGL-3 prohormone convertase.

7. It should be determined whether the 'client binding domain' of ENPL-1 is required for an interaction with DAF-28.

8. Figure 5B shows that IP of ASNA-1 does not co-precipitate tagged DAF-28. It isn't clear how to interpret such negative data, which might be better situated in a supplementary figure.

9. It would add value to the study to identify regions of ENLP-1 that are required for interaction with ASNA-1.

10. How the author's measured bypass of the asna-1 mutant phenotype by overexpression of ENLP-1 is not clear. The authors should measure coelomocyte fluorescence rather than assign samples to one of two categories.

11. It isn't clear why proteomics data are relegated to a supplementary figure.

12. This study, surprisingly, does not take advantage of the genetics of ILP signaling to determine whether ASNA-1 and ENLP-1 are required for DAF-28 function in vivo. For example, Cornils et al (PMID: 21343369) show effects of daf-28(lf) that could be used to test whether ASNA-1 and ENLP-1 are required for regulation of entry in dauer. The authors should consider adding genetic studies that put ASPN-1 and ENPL-1 in a pathway with DAF-28 and DAF-2/Insulin receptor.

© 2023. Published by The Company of Biologists under the terms of the Creative Commons Attribution License (https://creativecommons.org/licenses/by/4.0/).

13. The authors should more clearly discuss why DAF-28 is required for the interaction between ASNA-1 and ENLP-1. It is confusing that DAF-28 does not co-purify with ASNA-1 given that ENLP-1 binds DAF-28. The authors should consider incorporating a model in their manuscript.

Reviewer 2

Advance summary and potential significance to field

Podraza-Farhanieh et al. investigated the molecular mechanisms by which the ER chaperone ENPL-1/GRP94 and ASNA-1/TRC40/GET3 regulate the secretion of insulin-like peptides in C. elegans. Both were previously found to regulate the secretion of the insulin-like peptide DAF-28. In the current study, the authors demonstrated that ENPL-1 and ASNA-1 form a complex in a DAF-28-dependent manner.
This interaction is independent of the client-binding domain of ENPL-1, and is enhanced by oxidation of ASNA-1. The punctate distribution of ASNA-1 is altered in the enpl-1 mutant, and overexpression of ENPL-1 partially rescued the insulin secretion defects of the asna-1 mutant. These findings are potentially interesting and provide insights into the secretory mechanisms of insulin-like peptides. The significance of this study is further underscored by similar functions of the human homologs of these two genes in regulating insulin secretion from the pancreatic beta-cells. However, there are several concerns that need to be addressed, as detailed below.

Comments for the author

Major concerns:
1. The authors should provide some biological context for the observed interaction between ENPL-1 and ASNA-1. The author's group previously showed that the asna-1 mutant has a larval arrest phenotype, and that the enpl-1 shows growth defects with high level of ER stress. What happens in the asna-1; enpl-1 double mutant? If the double mutant is not viable, this question can still be addressed by using the auxin-mediated ASNA-1 knockdown to bypass potential embryonic lethality or early larval arrest.
2. Fig. 1B: The confocal microscopy does not convincingly show the colocalization of ENPL-1 and ASNA-1, as one could immediately tell the strikingly different subcellular distribution of the two molecules in ASI. The distribution of ENPL-1 is suggestive of an ER localization, whereas that of ASNA-1 is less clear. To demonstrate that ENPL-1 and ASNA-1 physically interact in the ASI neuron, the authors should consider experiments that show close proximity of ENPL-1 and ASNA-1 using split GFP, BiFC or PLA (proximity ligation assay). Alternatively, super-resolution microscopy should be considered.
3. Fig. 2B: Please show the BFP channel to indicate neurons, so that the decrease of ANSA-1::mNG signals could be evaluated. The reduction of ASNA-1::mNG signals also needs to be quantified. In this experiment, the authors reported treating the animals with auxin (N-KAA) for 48 hours, which seems unusually long for auxin-mediated protein knockdown. Please explain it.
4. Fig. 3A: The authors made an interesting observation that ENPL-1/ASNA-1 interaction is strengthened by DAF-28. It has been shown that increased ER stress response is observed in daf-28(sa191), suggesting that misfolding of DAF-28 causes ER stress (from Dennis Kim's lab). As the semi-dominant daf-28(sa191) mutant phenotype is hypothesized to result from interference of the mutant DAF-28 protein with other insulin-like peptides in the secretory pathway, could the authors examine ENPL-1/ASNA-1 interaction in daf-28(sa191) at the restrictive temperature (25 degree) as a further elaboration of DAF-28 dependence of ENPL-1/ASNA-1 interaction? In fact, the paper in its current form is purely mechanistic and does not involve any assays that demonstrate the biological or physiological significance of the observed results. The paper can be made more appealing by adding some flavor of physiology.
5. It is somewhat difficult to draw a conclusion from experiments in Figure 3B-3D. Results of the kpc-1 experiment are consistent with the notion that pro-DAF-28 enhances ENPL-1/ASNA-1 interaction. However, the effect of the ins-4 and unc-31 mutations are not adequately explained.
6. In the proteomic analysis (Fig. S4), the authors described upregulated proteins being enriched in the asna-1 mutant. Are these compensatory effects of blockade of insulin secretion in the asna-1 mutant? How about the downregulated proteins? If ASNA-1 is involved in Golgi-related trafficking, one would expect that important trafficking pathways are likely compromised in the asna-1 mutant. Please elaborate on this by analyzing the downregulated proteins.

© 2023. Published by The Company of Biologists under the terms of the Creative Commons Attribution License (https://creativecommons.org/licenses/by/4.0/).

7. Fig. 7: The localization of ASNA-1 to the Golgi needs to be verified by co-expression of a Golgi marker, such as AMAN-2 or SQV-8, with confocal microscopy.

Minor comments:
1. The Abstract needs to be written in a way that it appeals to more general readers from the beginning. Careful English editing is also advised to further polish the manuscript.
2. Please cite Brenner (Genetics, 1974) as a general reference for C. elegans cultivation.
3. The size labels of the western blot need to be better aligned to the bands in Fig 3A-3D and Fig. S2.
4. Fig. 1A: No GFP immunoreactivity was detected in the input for GFP pulldown of ENPL-1::FLAG/rab-3p::ASNA-1::GFP. Why? The original, uncropped blots should be provided so that the specificty of immunoreactivity could be evaluated.
5. Fig. 1C: Please show a close-up of the coelomocytes that contain DAF-28::mNeonGreen signals. Please also state clearly which coelomocytes (anterior middle, posterior) are scored in the DAF-28 secretion assays. In Fig. 1C and Fig. 2C, anterior coelomocytes are shown. In Fig. 6A, middle and posterior coelomocytes are shown. As DAF-28 is secreted from ASI and ASJ and is likely to form a gradient extracellularly, the location of the coelomocytes may affect the amount of DAF-28 that is endocytosed.

First revision

Author response to reviewers' comments

Reviewer 1 Comments for the Author:
The manuscript has several weaknesses that should be addressed.

1. Throughout the manuscript, summary data are presented as bar graphs. These should be replaced with scatter plots that clearly show the distribution of individual measurements.

2a. In several western blots there is a faint ENPL-1::FLAG signal under control conditions (anti-GFP pull-downs in the absence of ASPN-1::GFP). The authors should quantify ENPL-1::FLAG immunoreactivity to demonstrate the specificity of the ENPL-1::FLAG co-immunoprecipitation with ASPN-1::GFP

2b. The cellular expression of ASNA-1 is initially reported as restricted to or enriched in ASI and ASJ, neurons that produce the ILP DAF-28. Micrographs of an asna-1 reporter allele, however, suggest a much broader expression pattern (Figure 2). Claims of specific expression should be tempered and the authors should clarify their description of which cells express ANSA-1.

3. The authors state that ASNA-1 and ENPL-1 co-localize in neurons but the expression shown in Figure 1B does not clearly show co-localization. The degree of co-localization should be measured (the R2 value included in the figure is not clearly explained) and an appropriate negative control should be used.

4. In Figure 2 the authors label a transgene that drives expression of a component of the auxin-induced degradation system as 'reSi7' - this will not mean anything to most readers and the authors should use a label that informs the reader about the function of the transgene.

5. The authors show that mutation of genes required for ILP processing and release affect the interaction between ASPN-1 and ENPL-1. They also test whether mutation of another ILP, INS-4, affects this interaction and find that in ins-4 mutants there is increased association of ASPN-1 and ENPL-1. The rationale for this experiment is not clear, nor is it clear why loss of INS-4 affects the ASPN-1/ENPL-1 interaction.

6. p. 7 line 14 - 'cleaved by the EGL-3' should be 'cleaved by the EGL-3 prohormone convertase.

© 2023. Published by The Company of Biologists under the terms of the Creative Commons Attribution License (https://creativecommons.org/licenses/by/4.0/).

7. It should be determined whether the 'client binding domain' of ENPL-1 is required for an interaction with DAF-28.

8. Figure 5B shows that IP of ASNA-1 does not co-precipitate tagged DAF-28. It isn't clear how to interpret such negative data, which might be better situated in a supplementary figure.

9. It would add value to the study to identify regions of ENLP-1 that are required for interaction with ASNA-1.

10. How the author's measured bypass of the asna-1 mutant phenotype by overexpression of ENLP-1 is not clear. The authors should measure coelomocyte fluorescence rather than assign samples to one of two categories.

11. It isn't clear why proteomics data are relegated to a supplementary figure.

12. This study, surprisingly, does not take advantage of the genetics of ILP signaling to determine whether ASNA-1 and ENLP-1 are required for DAF-28 function in vivo. For example, Cornils et al (PMID: 21343369) show effects of daf-28(lf) that could be used to test whether ASNA-1 and ENLP-1 are required for regulation of entry in dauer. The authors should consider adding genetic studies that put ASPN-1 and ENPL-1 in a pathway with DAF-28 and DAF-2/Insulin receptor.

13. The authors should more clearly discuss why DAF-28 is required for the interaction between ASNA-1 and ENLP-1. It is confusing that DAF-28 does not co-purify with ASNA-1 given that ENLP-1 binds DAF-28. The authors should consider incorporating a model in their manuscript.

Reviewer 1 response:
1. We changed all graphs to scatter plots to clearly show the distribution of individual measurements **(Fig. 1D, Fig. 3B, Fig. 4A-D, Fig. 5A-B, Fig. 6B, Fig. S1A-D)**.

2. We performed western blots of lysates from wild-type, 3xFlag::ENPL-1 worms and ASNA-1::GFP worms and analyzed them using anti:Flag, anti:GFP and anti-tubulin (loading control) to show the specificity of the antibodies. The antibodies are specific since we do not detect 3XFlag::ENPL-1 with anti-GFP antibodies and ASNA-1::GFP with anti-Flag antibodies. The difference in molecular weights of the two tagged proteins allows us to unambiguously make this determination (**Fig. S3B**).

2b. ASNA-1::mNeonGreen::AID is indeed widely expressed in the whole animal. We added a representative picture that shows the expression of the protein and we added a description in the text (**Fig. 2A**).

3. We agree with the reviewer's comment. The "co-localization" word is too specific and difficult to address with the confocal experiments. The confocal picture is an addition to the Co-immunoprecipitation experiments which show the interaction of two proteins. We think that the appropriate word is "co-expression in the same cell". We changed the text to reflect that in the text. We note that our findings indicate that the interaction between ASNA-1 and ENPL-1 most likely to occur in the Golgi. Given the broad localization of both ASNA-1 and ENPl-1 within each cell, it is challenging to distinguish the specific interaction signal in the Golgi ministacks of the ASI neurons. We have tried to perform FRET with ASNA-1::GFP and ENPL-1::mKate2 but could not detect a convincing signal. This may be also in part because GFP and mKate2 are not an ideal FRET pair. However, we are confident that the interaction we detect by co-immunoprecipitation is real and biologically meaningful because it is reduced in *daf-28* deletion mutants as well as in worms ablated for the ASI neurons (where DAF-28 is expressed). Consistently most of the interaction is seen when ASNA-1::GFP expression is solely in neurons and the interaction is greatly increased in *kpc-1* mutants where DAF-28 accumulates in the pro-form and in conditions where ASNA-1 preferentially exists in the oxidized state (H202 treatment and *sod-2* mutants). Further expression of ENPL-1 and ASNA-1 in separate tissues led to no co-immuno-precipitation signal. Given the sensitivity and specificity of the co-IP signal to different conditions in both directions, we are confident that changes with the genetic background in predictable ways. Importantly when tagged ENPL-1 and ASNA-1 are expressed in different tissues in the same strain (neurons and muscle) no co-

© 2023. Published by The Company of Biologists under the terms of the Creative Commons Attribution License (https://creativecommons.org/licenses/by/4.0/).

immunoprecipitation signal is detected. We hope that this set of results convinces the reviewers that the interaction being observed is biologically meaningful.

4. We changed the information in figure 2 as requested (**new Fig. 3A-B**).

5. The rationale for this experiment was to ask if the interaction between ASNA-1 and ENPl-1 is specific for the DAF-28/ILP or is it more general for other insulin like peptides. We used *ins-4* mutants to ask whether interaction is specific for DAF-28/ILP because INS-4 and DAF-28 belong to the same (Beta) class of ILPs and both are DAF-2 agonists. That was the reason that we chose the *ins- 4* mutant for our test. Perhaps the writing around this was not clear and we have tried to improve the writing. As to why there is increased interaction between ASNA-1 and ENPL-1, that was a surprise to us. We speculate that the expression of *DAF-28::GFP* might be increased in *ins-4* mutants. However since the DAF-28:GFP expressing transgene (*svIs69)* and *ins-4* map quite close together we have not obtained the *ins-4(tm3620); svIs69* worms to look directly at the DAF-28 protein levels.

6. We added the writing about prohormone convertase in all the missing places.

7. We have shown this result in our previous publication in Development 2020 Oct 27;147(20):dev190082. doi: 10.1242/dev.190082. There we found that the binding of DAF-28/ILP to ENPL-1 required the client binding domain.

8. We have moved this data to a supplementary figure (**Fig. S8B**).

9. In would be definitely a valuable finding, but in most cases, the deletion of other regions of GRP94 (the mammalian homolog) leads to an unfolded/protein which makes it impossible to make meaningful conclusions about domain requirements. That is not a case of deletion of the client binding domain, because this deletion of it does not affect the protein structure and the ATPase activity.

10. We measured the fluorescence of DAF-28::GFP in coelomocytes using ImageJ. DAF-28::GFP coelomocyte fluorescence in otherwise wild-type worms was the control and based on that we measured the percentage of coelomocyte fluorescence in the other strains (**Fig. 6A-B).**

11. We moved the proteomics data to the main figures as suggested (**Fig. 7A-D**).

12. We agree that the biological analysis is very meaningful. However, we wish to note that in earlier papers we have characterized the role of ASNA-1 PMID: 17289575 and PMID: 33883621 and of ENPL-1 (PMID: 33037039) in IIS signaling we studied the physiological/biological roles of both *asna-1* and *enpl-1*. In these papers, we have shown that both ASNA-1 and ENPL-1 act to promote signaling through that IIS pathway to promote growth and that in sensitized mutant backgrounds both mutants show an enhanced constitutive dauer (Daf-c) phenotype. ASNA-1 acts non-autonomously to control growth. also overexpression of ASNA-1 (like DAF-28 and INS-4) promotes the escape from the dauer state of *daf-7* mutants raised at 25C. Both proteins have been shown to be required for DAF- 28/ILP secretion overexpression of ENPL- 1 leads to increased DAF-28/ILP secretion. ENPL-1 was shown to bind to DAF-28/ILP via the CBD domain and is required for its processing from the "pro DAF-28" form to the mature processed form. The oxidized form of ASNA-1 was shown to be the molecular form that acts in the promotion of DAF-28/ILP secretion. We do agree that the results in this manuscript have a more mechanistic flavor but that is due to the fact that we have published on the cell biology of protein action in earlier publications.

13. Our model to describe the action of ENPL-1, DAF-28 and ASNA-1 builds on our findings and of findings in previous publications from our lab and other labs. We favor the model by which ENPL-1 binds to DAF-28/ILP in the ER and takes it to the Golgi where DAF-28 is transferred to the sites of dense core vesicles budding so that DAF-28 is incorporated into DCVs for processing and secretion. ENPL-1 that has released the DAF-28 client interacts with ASNA-1 in the Golgi and ASNA-1 promotes its retrograde transport back to the ER where it can acquire new DAF-28. In a *daf-28* mutant ENPL-1, lacking it's client, will not move from the ER to the Golgi and back to the ER and in this case ASNA-1 will not interact with the cargo-free ENPL-1. In an *asna-1* mutant, this retrograde transport step to bring back ENPL-1 to the ER will not occur leading to defective DAF-28 secretion. It was to

© 2023. Published by The Company of Biologists under the terms of the Creative Commons Attribution License (https://creativecommons.org/licenses/by/4.0/).

test this notion that we examined the effect of overexpression of ENPL-1. The expectation was that increased levels of ENPL-1 will reduce the demand for it's retrograde transport back to the ER and be sufficient to promote some level of DAF-28 secretion. This is what we see in *asna-1* mutants overexpressing ENPL-1. We have added a description of the model in the discussion section.

We note that this model builds findings in yeast and mammalian systems showing that ASNA-1 homologs are involved in retrograde transport, oxidized yeast GET3 has chaperone function, mammalian GRP94 is found in the Golgi and is sent back to the ER via its C-terminal ER retrieval signal. Moreover, our own published findings show that ENPL-1 is found outside the ER and that oxidized ASNA-1 (the chaperone form) is likely responsible for promoting DAF-28 secretion.

Reviewer 2 Comments for the Author:
Major concerns:

1. *The authors should provide some biological context for the observed interaction between ENPL-1 and ASNA-1. The author's group previously showed that the asna-1 mutant has a larval arrest phenotype and that the enpl-1 shows growth defects with high level of ER stress. What happens in the asna-1; enpl-1 double mutant? If the double mutant is not viable, this question can still be addressed by using the auxin-mediated ASNA-1 knockdown to bypass potential embryonic lethality or early larval arrest.*

2. *Fig. 1B: The confocal microscopy does not convincingly show the colocalization of ENPL-1 and ASNA-1, as one could immediately tell the strikingly different subcellular distribution of the two molecules in ASI. The distribution of ENPL-1 is suggestive of an ER localization, whereas that of ASNA-1 is less clear. To demonstrate that ENPL-1 and ASNA-1 physically interact in the ASI neuron, the authors should consider experiments that show close proximity of ENPL-1 and ASNA-1 using split GFP, BiFC or PLA (proximity ligation assay). Alternatively, super-resolution microscopy should be considered.*

3. *Fig. 2B: Please show the BFP channel to indicate neurons, so that the decrease of ANSA-1::mNG signals could be evaluated. The reduction of ASNA-1::mNG signals also needs to be quantified. In this experiment, the authors reported treating the animals with auxin (N-KAA) for 48 hours, which seems unusually long for auxin-mediated protein knockdown. Please explain it.*

4. *Fig. 3A: The authors made an interesting observation that ENPL-1/ASNA-1 interaction is strengthened by DAF-28. It has been shown that increased ER stress response is observed in daf-28(sa191), suggesting that misfolding of DAF-28 causes ER stress (from Dennis Kim's lab). As the semi-dominant daf-28(sa191) mutant phenotype is hypothesized to result from interference of the mutant DAF-28 protein with other insulin-like peptides in the secretory pathway, could the authors examine ENPL-1/ASNA-1 interaction in daf-28(sa191) at the restrictive temperature (25 degree) as a further elaboration of DAF-28 dependence of ENPL-1/ASNA-1 interaction? In fact, the paper in its current form is purely mechanistic and does not involve any assays that demonstrate the biological or physiological significance of the observed results. The paper can be made more appealing by adding some flavor of physiology.*

5. *It is somewhat difficult to draw a conclusion from experiments in Figure 3B-3D. Results of the kpc- 1 experiment are consistent with the notion that pro-DAF-28 enhances ENPL-1/ASNA-1 interaction. However, the effect of the ins-4 and unc-31 mutations are not adequately explained.*

6. *In the proteomic analysis (Fig. S4), the authors described upregulated proteins being enriched in the asna-1 mutant. Are these compensatory effects of blockade of insulin secretion in the asna-1 mutant? How about the downregulated proteins? If ASNA-1 is involved in Golgi-related trafficking, one would expect that important trafficking pathways are likely compromised in the asna-1 mutant. Please elaborate on this by analyzing the downregulated proteins*

7. *Fig. 7: The localization of ASNA-1 to the Golgi needs to be verified by co-expression of a Golgi marker, such as AMAN-2 or SQV-8, with confocal microscopy*

© 2023. Published by The Company of Biologists under the terms of the Creative Commons Attribution License (https://creativecommons.org/licenses/by/4.0/).

Minor comments:
1. The Abstract needs to be written in a way that it appeals to more general readers from the beginning. Careful English editing is also advised to further polish the manuscript.
2. Please cite Brenner (Genetics, 1974) as a general reference for C. elegans cultivation.
3. The size labels of the western blot need to be better aligned to the bands in Fig 3A-3D and Fig. S2.
4. Fig. 1A: No GFP immunoreactivity was detected in the input for GFP pulldown of ENPL-1::FLAG/rab3p::ASNA-1::GFP. Why? The original, uncropped blots should be provided so that the specificity of immunoreactivity could be evaluated.
5. Fig. 1C: Please show a close-up of the coelomocytes that contain DAF-28::mNeonGreen signals. Please also state clearly which coelomocytes (anterior, middle, posterior) are scored in the DAF-28 secretion assays. In Fig. 1C and Fig. 2C, anterior coelomocytes are shown. In Fig. 6A, middle and posterior coelomocytes are shown. As DAF-28 is secreted from ASI and ASJ and is likely to form a gradient extracellularly, the location of the coelomocytes may affect the amount of DAF-28 that is endocytosed.

Reviewer 2 response (major concerns) :
1. The reviewer is correct in anticipating that pan-somatic depletion of ASNA-1::AID will lead to a 1st larval stage arrest. We have analyzed this phenotype in a paper accepted for publication recently by PLoS Genetics and currently available at Biorxiv (https://www.biorxiv.org/content/10.1101/2022.07.12.499739v1)

We have performed the analysis of *asna-1; enpl-1* double knockdown in two ways.

First, we constructed a *enpl-1(ok1964)/tmC5green balancer; asna-1(ok938)/oxTi719 unc-32(red balancer)*. *enpl-1;asna-1* double mutants segregating from this strain will be non-Green non-Red. We do not find any non-green non-Red larvae emerging from these mothers. This absence of class shows that the double mutants die as embryos. The data is presented in in the text.

Secondly, we constructed a strain to deplete ASNA-1::mNeonGreen::AID in all somatic cells using a pansomatic TIR1 driver and auxin treatment in an *enpl-1/balancer* background. From this strain, we exposed 4th larval stage *enpl-1/enpl-1* animals to auxin treatment in order to bypass the 1st larval stage arrest seen when *asna-1* is depleted in an *enpl-1(+)* background. We find that in contrast to non-auxin treated siblings, pan somatic ASNA-1 depletion in *enpl-1* mutants leads to a complete loss in egg laying ability (**Fig. S1D**).

2. As mentioned in our response to point 3 of reviewer#1, we have attempted FRET analysis without success. We feel that given the broad expression of both ENPL-1::mKate2 and ASNA-1::GFP within each cell, and the likely interaction within the Golgi ministacks, it will be very challenging to detect the transient interactions between the two proteins by microscopy methods and that a co-IP analysis is likely the best way to perform the interaction analysis. As detailed in our reply to the same question by reviewer #1 the various conditions under which the co-IP was performed in order to validate it will hopefully convince reviewer#2 that this interaction is biologically valid. We note also that we present evidence of suppression of the secretion defect of *asna-1* by ENPL-1overexpression as proof of their genetic interaction.

3. We added the analysis BFP channel to the analysis. We showed that in the control (no Auxin), there are many BFP positive neurons, while after auxin-mediated knock-down, there are none or at most one positive. This indicates that the auxin knockdown is not fully penetrant. We cannot quantify the knockdown of BFP using western blot because there are no available antibodies since the only company (Evrogen) making these antibodies is in Russia and delivery of goods from there is not possible at the present time. We do not know why the *reSi7* transgene for neuron-specific degradation does not work with great efficiency in neurons. It might partially have to do with our use of the water- soluble K-NAA rather than the ethanol-soluble indole acetic acid which in some cases induces a stronger phenotype (PMID: 33677541) (**Fig. 3A**).

4. While it is true that *sa191* mutants have increased ER stress (as shown by work from the Dennis Kim lab) perhaps by unfolded DAF-28, we have shown in our 2020 paper in *Development* that reducing ER stress in neurons by expressing spliced XBP-1 does not suppress the secretion phenotype

© 2023. Published by The Company of Biologists under the terms of the Creative Commons Attribution License (https://creativecommons.org/licenses/by/4.0/).

of *enpl-1* mutants. The analysis of the *daf-28(sa191)* mutant by the Gidalevitz lab (PMID: 27926939) shows convincingly that the dauer phenotype of *sa191* is due to a combination of defects in the IIS pathway and the DAF-7/TGFbeta pathway. The DAF-7 pathway activity is low in *sa191* mutants and forced expression of DAF-7 ameliorates the *sa191* phenotype. This effect on lowered DAF-7 pathway is not seen in *daf-7(tm2308)* deletion mutants. Thus the combined deficit TGFbeta and IIS signaling seen in *sa191* mutants indicates to us that using the *sa191* mutant (as an additional means to reduce DAF-28 secretion) to further show the dependence of the ENPL-1/ASNA-1 on DAF-28 will be problematic. The work on *sa191* (PMID: 27926939) also presented evidence that secretion of DAF-28 was likely not affected by the R37C mutation in *daf-28(sa191)*.

This is consistent with the analysis of the Gidalevitz group and shows that the *sa191* mutant cannot be used to examine the consequences of a deficit in DAF-28 secretion. We are presenting the secretion data of DAF- 28^{R37C}::mNeonGreen in the response letter for your evaluation since we do not know how to fit the narrative of why we cannot use DAF-28^{R37C}::mNeonGreen in the text of the manuscript.

Note: We have removed data that was provided for the referees in confidence.

5. We have added more explanation for the effects of *unc-31* in the discussion. To summarize: the *ins- 4* mutant was used to ask whether the DAF-28 dependency of the ENPL-1/ASNA-1 interaction was specific to that ILP or more general in nature. In *unc-31* mutant, neuropeptides like DAF-28 are thought to be processed and packaged into mature DCVs that are not primed for release. This provides a condition where (in contrast to *kpc-1* mutants) the DAF-28 protein will be largely in the mature processed form and allow us to ask what the effect on the ASNA-1/ENPL-1 interaction will be when there are low levels of the pro-DAF-28 form. We have an explanation for the reason we used *ins-4* mutants in our analysis in the discission. We hope that this will adequately explain our motivation.

6. We apologize for the insufficiently explained results. The proteomic analysis we presented here shows enriched pathways which have been found in the *asna-1(ok938)* mutant compared to N2 control. To address that we uploaded all of the information we have regarding the analysis. This includes:

a) list of all detected proteins in the proteomic analysis,
b) list of significantly changed proteins in *asna-1(ok938)* mutants
c) Reactome enriched pathways data with proteins that belong to the groups with information which of them are up and down-regulated in *asna-1* mutants.
d) List of top 20 most upregulated and bottom 20 most downregulated proteins in the *asna- 1(ok938)* mutants.
e) Additionally, we changed the information in the text and added missing information. **(Fig. 7A-D, Table S1)**.

7. We have built a plasmid containing Posm-6::AMAN2::wrmScarlet and used it to generate the transgene *rawEx90* and expressed it in the ASNA-1::mNeonGreen background. We found that strong ASNA-1::mNeonGreen puncta corresponds with the AMAN-2::wrmScarlet (**Fig. 8B)**.

Reviewer#2 (Minor comments):

1. We have a new version of the abstract that we hope will be more suitable. We have also tried to improve our writing.
2. We cited Brenner (Genetics, 1974).
3. We aligned the size labels of the western blot to the bands in all figures (**Fig. 1D, Fig. 4A-D, Fig. 5A-B, Fig. S1A, Fig. S2A, B, Fig. S3A-B, Fig. S5 A-B, Fig. S6A-B, Fig. S7A-B, Fig. S8A-B).**
4. In figure 1A, ASNA-1::GFP expressed under the pRAB3 promoter is only expressed in a neurons and this is not sufficient for the signal to be detected from the 10ug of total protein lysate. The pull-down of ASNA-1::GFP in this strain shows however that there is expression, but it is less than when ASNA-1::GFP is expressed under its own promoter.

We included all of the uncropped western blots (**Fig. S2A-B, Fig. S3A-B, Fig. S5 A-B, Fig. S6A-B, Fig. S7A-B, Fig. S8A-B).**

© 2023. Published by The Company of Biologists under the terms of the Creative Commons Attribution License (https://creativecommons.org/licenses/by/4.0/).

5. We added the close-up of the coelomocyte that contains DAF-28::mNeonGreen signals and stated that it is an anterior coelomocyte (**Fig. 1C**). We added this information to all of the figures that represent the coelomocytes (**Fig. 3B, Fig. 6A**).

Second decision letter

MS ID#: DEVELOP/2022/201035

MS TITLE: Proinsulin dependent interaction between ENPL-1/GRP94 and ASNA-1 in neurons is required to maintain insulin secretion in C. elegans.

AUTHORS: Agnieszka Podraza-Farhanieh, Dorota Raj, Gautam Kao, and Peter Naredi

I have now received all the referees reports on the above manuscript, and have reached a decision. The overall evaluation is positive and we would like to publish a revised manuscript in Development, however, the referees' provide recommendations that add further clarity to the manuscript. I do not expect to send this back to the reviewers, however, please do take care to address each point in the revised manuscript. Please attend to all of the reviewers' comments in your revised manuscript and detail them in your point-by-point response. If you do not agree with any of their criticisms or suggestions explain clearly why this is so.

Reviewer 1

Advance summary and potential significance to field

Podraza-Farhanieh and colleagues show that an interaction between the ER chaperone ENPL-1 and a conserved membrane trafficking factor ASNA-1 is required for secretion of insulin-like peptides in C. elegans. These results identify components of a membrane trafficking mechanism that is required for insulin signaling in vivo and that is likely conserved between nematodes and vertebrates.

Comments for the author

The authors provided detailed responses to questions raised during review of the original manuscript. The revised manuscript addresses those questions. I have only one minor comment: in more than one instance the authors state that unc-31 mutants have increased numbers of mature DCVs compared to the wild type but this statement is not clearly references. Please make clear which published work supports this statement - I believe it is Hammarlund et al. 2008.

Reviewer 2

Advance summary and potential significance to field

The authors describe a pair of organelle trafficking proteins that physically interact to regulate the secretion of an insulin-like peptide from nematode neurons. As neuropeptides, including insulin and insulin-like peptides, are critical regulators or animal physiology, development and metabolism, this study provides insights into the molecular mechanisms by which these chaperone and trafficking proteins regulate neuropeptide secretion.

Comments for the author

The authors had addressed most of the concerns that I raised in the initial review of the manuscript with experiments or revision of the manuscript. However, the revised manuscript still shows a few problems that need to be fixed:

© 2023. Published by The Company of Biologists under the terms of the Creative Commons Attribution License (https://creativecommons.org/licenses/by/4.0/).

1. Fig. 1B: Please indicate clearly what this neuron is, both in the text and the figure legend. In the text, the authors stated "ASNA-1 and ENPL-1 expression was found in overlapping neurons based on confocal microscopy of worms coexpressing ASNA-1::GFP and ENPL-1::mKate2". But Fig. 1B shows only ONE neuron. The authors need to provide other images to support their claim that these two proteins are present in several neurons, or they should modify their statement and clearly indicate which neuron it is in Fig. 1B.

2. In the new Fig. 3A, AID is also tagged on ANSA-1::mNG. Therefore, after auxin treatment, one expects to see reduction in the fluorescence not only in BFP (from reSi7: Pregf-1::TIR-1::F2A::BFP::AID), but also in green fluorescence (mNG). The reduction of ANSA-1::mNG::AID specifically in neurons is necessary to support effectively knockdown of ANSA-1. Please provide this data. Also in Fig. 3A and the legend, please clearly indicate “Neuronal TIR-1::F2A::BFP::AID, so that the readers understand where the BFP signals come from.

3. I appreciate the authors’ effort to perform the DAF-28(R37C) secretion assay. I just want to point out that fluorescence tag at the C-terminus of the neuropeptides is usually cleaved during the process of peptide maturation. Therefore, the green fluorescence in the coelomocytes may represent just the mNG protein, although the peptide sequence of DAF-28 lacks the typical dibasic motifs (KR or RK) that the EGL-3 endopeptidase recognizes to perform the cleavage, so one may assume that the fluorescence signal in the coelomocytes represents DAF-28(R37C)::mNG protein, not the free form mNG protein. Given the mNG epitope is much larger than DAF-28, this approach may still be somewhat problematic. But I am fine with what the authors had done so far and there is no need to address this issue further.

4. The top 20 up- or downregulated protein list is not very informative, as most of them are uncharacterized proteins.

5. I feel that the prior version of the abstract is better than the new one. The first sentence of the abstract should provide a general context for the entire study. For example, something like “Neuropeptides, including insulin, are important regulators of physiological functions of the organisms. The Golgi trafficking pathway is a critical mechanism that regulates the secretion of insulin-like peptides. Here we show that …” The first paragraph of Introduction is also very specific, which may present as a challenge for readers that are not in this field. I suggest that the authors describe some general backgrounds for insulin secretion not just for C. elegans, but for mammals as well. Some editing of the manuscript is also strongly recommended - for example, some punctuations are missing, making many descriptions unnecessarily lengthy. Number the lines so that the reviewer can point out specific lines that need revision.

6. The authors are recommended to provide a list of strains used in this study that clearly shows the strain names and genotype information following the standard C. elegans nomenclature system. This will make it convenient for the readers to understand the genetics of this study and for strain request to be made in the future.

7. Page 5, starting from the end of line 2: "... and the protein was secreted into the coelomocytes..." This is a misleading statement, since the protein is secreted into the pseudocoelom, from which the coelomocytes uptake it via pinocytosis or endocytosis. This needs to be corrected.

8. Fig. 2B: The label should be "Prab-3::NLS::RFP", to be exact.

9. The last line of page 5: "pro-form" does not sound like a standard terminology. Maybe "DAF-28 pro-peptide" is more appropriate.

10. In the unc-31 mutant, not only DAF-28, but many other neuropeptides are prevented from secretion. Therefore, the results of ASNA-1/ENPL-1 interaction in the unc-31 mutant cannot be interpreted as a result of increased DAF-28 level. If the authors would like to draw such a conclusion, the experiment to do is DAF-28 overexpression. Otherwise, they should tune down this interpretation and leave other possibilities open.

© 2023. Published by The Company of Biologists under the terms of the Creative Commons Attribution License (https://creativecommons.org/licenses/by/4.0/).

11. The observation that ENPL-1 overexpression bypasses the requirement of ASNA-1 is hard to reconcile with the data that ENPL-1 physically forms a complex with ASNA-1. The authors imply that excess ENPL-1 can compensate for the loss of ASNA-1. I think this makes it somewhat ambiguous what the significance is for ASNA-1/ENPL-1 interaction. In Discussion, the authors speculate that this is because the retrograde step (I think they mean Golgi-to-ER trafficking of ENPL-1?) is less important, but this speculation should be cautioned, as retromer complexes - proteins required for this retrograde step - are critical proteins and have been shown to be indispensable in cellular secretion and vesicular trafficking. So there is no reason to believe that this retrograde process of ENPL-1-related trafficking is not as important as the one that targets DAF-28 for secretion. I suggest the authors to modify and tune down this statement.

12. The legends of Fig. 2 and Fig. 7 are very short, especially for Figure 7. Please provide more information in the legends to help the readers understand these figures better.

Second revision

Author response to reviewers' comments

Reviewer 1 Comments for the author

The authors provided detailed responses to questions raised during review of the original manuscript. The revised manuscript addresses those questions. I have only one minor comment: in more than one instance the authors state that unc-31 mutants have increased numbers of mature DCVs compared to the wild type but this statement is not clearly references. Please make clear which published work supports this statement - I believe it is Hammarlund et al. 2008.

- We addressed this comment and added Hammarlund et al. 2008 in the References to the text.

Reviewer 2 Comments for the author

The authors had addressed most of the concerns that I raised in the initial review of the manuscript with experiments or revision of the manuscript. However, the revised manuscript still shows a few problems that need to be fixed:

1. Fig. 1B: Please indicate clearly what this neuron is, both in the text and the figure legend. In the text, the authors stated "ASNA-1 and ENPL-1 expression was found in overlapping neurons based on confocal microscopy of worms coexpressing ASNA-1::GFP and ENPL-1::mKate2". But Fig. 1B shows only ONE neuron. The authors need to provide other images to support their claim that these two proteins are present in several neurons, or they should modify their statement and clearly indicate which neuron it is in Fig. 1B.
- We showed in Kao et al 2007 (PMID: 17289575) that ASNA-1::GFP is expressed in ASI and ASK head neurons. Additionally, in Podraza-Farhanieh et al 2020 (PMID: 33037039) we showed that ENPL-1 is expressed in many head neurons. We have added the missing information both in the result and figure legend sections of the manuscript. We have added a new figure (Fig 1B) from worms co-expressing ENPL-1::mKate2 and ASNA-1::GFP to show that ENPL-1::mKate2 is detected in the nerve ring and ASNA-1::GFP in two neurons is seen in that location. We have also indicated other tissues where the two proteins are detected.

2. In the new Fig. 3A, AID is also tagged on ANSA-1::mNG. Therefore, after auxin treatment, one expects to see reduction in the fluorescence not only in BFP (from reSi7: Pregf-1::TIR-1::F2A::BFP::AID), but also in green fluorescence (mNG). The reduction of ANSA-1::mNG::AID specifically in neurons is necessary to support effectively knockdown of ANSA-1. Please provide this data. Also in Fig. 3A and the legend, please clearly indicate “Neuronal TIR-1::F2A::BFP::AID, so that the readers understand where the BFP signals com
- We have added a photomicrograph showing that ASNA-1::mNeonGreen fluorescence is depleted in the nerve ring region following auxin treatment. This is shown in the new Fig 3A with

© 2023. Published by The Company of Biologists under the terms of the Creative Commons Attribution License (https://creativecommons.org/licenses/by/4.0/).

the white arrow indicating the region of the nerve ring where ASNA-1::mNeonGreen is no longer detected while expression is still preserved in the pharynx and the intestine.
- Secondly, we added the missing information to the figure legend “ASNA-1::mNG::AID;neuronal TiR1:: F2A::BFP:: AID” to indicate why BFP is depleted with auxin exposure. We have also added this information in the text of the results section. We hope this will be clear now.

3. I appreciate the authors’ effort to perform the DAF-28(R37C) secretion assay. I just want to point out that fluorescence tag at the C-terminus of the neuropeptides is usually cleaved during the process of peptide maturation. Therefore, the green fluorescence in the coelomocytes may represent just the mNG protein, although the peptide sequence of DAF-28 lacks the typical dibasic motifs (KR or RK) that the EGL-3 endopeptidase recognizes to perform the cleavage, so one may assume that the fluorescence signal in the coelomocytes represents DAF-28(R37C)::mNG protein, not the free form mNG protein. Given the mNG epitope is much larger than DAF-28, this approach may still be somewhat problematic. But I am fine with what the authors had done so far and there is no need to address this issue further.
- Thank you.

4. The top 20 up- or downregulated protein list is not very informative, as most of them are uncharacterized proteins.
- We added this information based on the previous review comments. But we agree that the list does not provide as much information as we had hoped.

5. I feel that the prior version of the abstract is better than the new one. The first sentence of the abstract should provide a general context for the entire study. For example, something like “Neuropeptides, including insulin, are important regulators of physiological functions of the organisms. The Golgi trafficking pathway is a critical mechanism that regulates the secretion of insulin-like peptides. Here we show that …” The first paragraph of Introduction is also very specific, which may present as a challenge for readers that are not in this field. I suggest that the authors describe some general backgrounds for insulin secretion not just for C. elegans, but for mammals as well. Some editing of the manuscript is also strongly recommended - for example, some punctuations are missing, making many descriptions unnecessarily lengthy. Number the lines so that the reviewer can point out specific lines that need revision.
- We edited the manuscript accordingly. We changed the abstract, as suggested by the reviewer, adding more general sentences. We hope this version of the abstract will be more suitable.
- We have started the introduction with a more general statement. We have edited the manuscript to improve the readability and added line numbers.

6. The authors are recommended to provide a list of strains used in this study that clearly shows the strain names and genotype information following the standard C. elegans nomenclature system. This will make it convenient for the readers to understand the genetics of this study and for strain request to be made in the future.
- Table S2 contains list of all the strains used in this study with the genotype and additional description.

7. Page 5, starting from the end of line 2: "... and the protein was secreted into the coelomocytes..." This is a misleading statement, since the protein is secreted into the pseudocoelom, from which the coelomocytes uptake it via pinocytosis or endocytosis. This needs to be corrected.
- Thank you for pointing that out. We changed the indicated sentence and others where the same type of mistake occurred.

8. Fig. 2B: The label should be "Prab-3::NLS::RFP", to be exact.
- Thank you for the comment. We changed it accordingly. We note that the fluorescent protein is TagRFP in the otIs356 expressing worms and have made the changes.

9. The last line of page 5: "pro-form" does not sound like a standard terminology. Maybe "DAF-28 pro-peptide" is more appropriate.

© 2023. Published by The Company of Biologists under the terms of the Creative Commons Attribution License (https://creativecommons.org/licenses/by/4.0/).

- Thank you for the comment. We changed to the DAF-28 pro-peptide as suggested throughout the text.

10. In the unc-31 mutant, not only DAF-28, but many other neuropeptides are prevented from secretion. Therefore, the results of ASNA-1/ENPL-1 interaction in the unc-31 mutant cannot be interpreted as a result of increased DAF-28 level. If the authors would like to draw such a conclusion, the experiment to do is DAF-28 overexpression. Otherwise, they should tune down this interpretation and leave other possibilities open.
- We tuned down the interpretation in the discussion section as requested.

11. The observation that ENPL-1 overexpression bypasses the requirement of ASNA-1 is hard to reconcile with the data that ENPL-1 physically forms a complex with ASNA-1. The authors imply that excess ENPL-1 can compensate for the loss of ASNA-1. I think this makes it somewhat ambiguous what the significance is for ASNA-1/ENPL-1 interaction. In Discussion, the authors speculate that this is because the retrograde step (I think they mean Golgi-to-ER trafficking of ENPL-1?) is less important, but this speculation should be cautioned, as retromer complexes - proteins required for this retrograde step - are critical proteins and have been shown to be indispensable in cellular secretion and vesicular trafficking. So there is no reason to believe that this retrograde process of ENPL-1-related trafficking is not as important as the one that targets DAF-28 for secretion. I suggest the authors to modify and tune down this statement.
- We modified and tuned down the statement in the discussion section as requested.

12. The legends of Fig. 2 and Fig. 7 are very short, especially for Figure 7. Please provide more information in the legends to help the readers understand these figures better.
- We added more information to the indicated figure legends.

Third decision letter

MS ID#: DEVELOP/2022/201035

MS TITLE: Proinsulin dependent interaction between ENPL-1/GRP94 and ASNA-1 in neurons is required to maintain insulin secretion in C. elegans.

AUTHORS: Agnieszka Podraza-Farhanieh, Dorota Raj, Gautam Kao, and Peter Naredi
ARTICLE TYPE: Research Article

I am happy to tell you that your manuscript has been accepted for publication in Development, pending our standard ethics checks.

© 2023. Published by The Company of Biologists under the terms of the Creative Commons Attribution License (https://creativecommons.org/licenses/by/4.0/).
